# HIV-1 matrix protein p17 misfolding forms toxic amyloidogenic assemblies that induce neurocognitive disorders

**DOI:** 10.1038/s41598-017-10875-0

**Published:** 2017-09-04

**Authors:** Yasmin Zeinolabediny, Francesca Caccuri, Laura Colombo, Federica Morelli, Margherita Romeo, Alessandro Rossi, Silvia Schiarea, Carlotta Ciaramelli, Cristina Airoldi, Ria Weston, Liu Donghui, Jerzy Krupinski, Rubén Corpas, Elisa García-Lara, Sara Sarroca, Coral Sanfeliu, Mark Slevin, Arnaldo Caruso, Mario Salmona, Luisa Diomede

**Affiliations:** 1 School of Healthcare Science, John Dalton Building, Manchester Metropolitan University, Chester Street, Manchester, M1 5GD UK; 2 Department of Molecular and Translational Medicine, University of Brescia, Piazza del Mercato 15, 25121 Brescia, Italy; 3 Department of Molecular Biochemistry and Pharmacology, IRCCS- Istituto di Ricerche Farmacologiche “Mario Negri”, Via G. La Masa 19, 20156 Milano, Italy; 4 Department of Environmental Health Sciences, IRCCS- Istituto di Ricerche Farmacologiche “Mario Negri”, Via G. La Masa 19, 20156 Milano, Italy; 5 Department of Biotechnologies and Biosciences, University of Milano Bicocca, Piazza dell’Ateneo Nuovo 1, 20126 Milano, Italy; 6 Hospital Universitari Mútua de Terrassa, Department of Neurology, Terrassa, Barcelona Spain; 7 Institut d’Investigaciones Biomèdiques de Barcelona, CSIC and IDIBAPS, Barcelona, Spain; 8 University of Medicine and Pharmacy, Targu Mures, Romania; 9 Department of Pathology/Medicine, Griffith University, Brisbane, Australia

## Abstract

Human immunodeficiency virus type-1 (HIV-1)-associated neurocognitive disorder (HAND) remains an important neurological manifestation that adversely affects a patient’s quality of life. HIV-1 matrix protein p17 (p17) has been detected in autoptic brain tissue of HAND individuals who presented early with severe AIDS encephalopathy. We hypothesised that the ability of p17 to misfold may result in the generation of toxic assemblies in the brain and may be relevant for HAND pathogenesis. A multidisciplinary integrated approach has been applied to determine the ability of p17 to form soluble amyloidogenic assemblies *in vitro*. To provide new information into the potential pathogenic role of soluble p17 species in HAND, their toxicological capability was evaluated *in vivo*. In *C*. *elegans*, capable of recognising toxic assemblies of amyloidogenic proteins, p17 induces a specific toxic effect which can be counteracted by tetracyclines, drugs able to hinder the formation of large oligomers and consequently amyloid fibrils. The intrahippocampal injection of p17 in mice reduces their cognitive function and induces behavioral deficiencies. These findings offer a new way of thinking about the possible cause of neurodegeneration in HIV-1-seropositive patients, which engages the ability of p17 to form soluble toxic assemblies.

## Introduction

The advent of combined antiretroviral therapy (cART) has converted the course of human immunodeficiency virus type-1 (HIV-1) from an incurable disease to a treatable chronic disease. As a result, the scientific community has shifted its attention from treating the acute complications of the disease to managing the long-term complications^1^. HIV-1-associated neurocognitive disorder (HAND) is one of the most frequent complications in HIV-1-seropositive (HIV^+^) patients and may result in the development of neurological deficiencies^1^. Disruption of neurocognitive functions in HIV^+^ patients adversely affects the quality of life, treatment adherence and lifespan of these patients. While the introduction of cART has completely changed the perspective of HAND and resulted in an important reduction of dementia, a high frequency of neurocognitive disorders persist^2^. The pathogenesis of HAND is unknown, even though damage and death of neuronal cells has been associated with the development of neurocognitive impairment^1^. Many mechanisms have been proposed as contributing factors to HAND, including induction of oxidative stress in the central nervous system (CNS), chronic microglial-mediated neuroinflammation, amyloid-beta (Aβ) deposition, hyperphosphorylated tau protein, and toxic effects of cART^1, 2^. However up to date, the pathological cause of cognitive decline in HIV^+^ patients under successful cART is not fully understood.

While in the pre-cART era the plasma viral load predicted the development of HAND, this is no longer true for the cART era. However, there is a persistent state of low-level virus replication in the cerebrospinal fluid (CSF), with evidence of immune activation, indicating the brain as a viral reservoir^3^. Many authors report a positive correlation between the amount of virus and viral products (e.g., gp120 and gp41) and the extent of histopathologic changes in the brain^4^. Therefore, a role of HIV-1 in HAND during cART cannot be completely ruled out. More recently, the inflammatory microenvironment, as a potential contributor to HAND, has become an important field of research. The degree of neurologic dysfunction was strongly related to the extent of macrophage activation on histopathology^5^, with production of inflammatory cytokines and chemokines, such as tumor necrosis factor-α and monocyte chemoattractant protein-1^6^. This chronic inflammation is probably exacerbated by HIV-1 proteins expressed in latently infected cells or in cells sustaining low-level HIV-1 replication, which further activate inflammatory pathways, leading directly to neural toxicity^7–10^. This evidence suggests that HAND with viral suppression may be driven by viral components such as Negative Regulatory Factor, Tat and Viral protein R, while HAND without or with partial viral suppression may also be fueled by whole virus, particularly envelope proteins^4^.

One possibility contributing to HAND include the presence of low level viremia in the CNS that may chronically drive neurodegeneration, although neurons are refractory to HIV-1 infection. It has been supposed that pathophysiological changes accompanying HAND include alterations in the function of HIV-1 infected astrocytes, macrophages and microglia cells. Indeed, upon HIV-1 infection, these cells secrete several neurotoxic factors leading to endothelial cell (EC) apoptosis and dysfunctional autophagy of neurons^11^.

Another possible explanation is the release of toxic viral products either in blood or in the CNS by latently HIV-1-infected cells that may trigger activation and aberrant functioning of different cells in the brain, including ECs, thus promoting inflammation and neurodegeneration.

The HIV-1 matrix protein p17 (p17) is a 132 amino acid (aa) structural protein. In mature virions p17 is myristoylated at N-terminus (myr-p17) and forms a protective shell that lines the inner leaflet of the viral membrane, although it can dissociate from the lining to direct the pre-integration complex to the host cell nucleus and to perform crucial functions throughout the HIV-1 life cycle^12^. The viral protein is continuously released into the extracellular space from HIV-1-infected cells, and can be detected in the plasma of HIV^+^ patients and in tissue specimens^11^. Moreover, p17 accumulates and persists in different organs and tissues of patients under cART, even in the absence of any replicative activity^13–15^. Several reports have shown that HIV-1 transcription can be efficiently induced by different stimuli^16^, even in the presence of protease inhibitors^17^. All these findings strongly suggest that p17 may be chronically present in the infected microenvironment, even during pharmacological control of viral replication. Extracellularly, p17 has been found to deregulate the biological activity of many different immune cells^18^, and to exert chemokine^19, 20^, pro-angiogenic^13^ and lymphangiogenic^21^ activities. All activities of p17 occur after interaction between the functional epitope (AT20) located at the N-terminal region of the viral protein^22^ and cellular receptors expressed on different target cells^13, 20, 23^. Indeed, antibodies to AT20 blocked p17/receptor interaction and all p17 biological activities^22, 24^.

The ability of a protein to misfold, particularly to change conformation from an α-helix to β-sheet, is a typical characteristic of amyloidogenic proteins actively involved in the pathogenesis of many human diseases, including some neurodegenerative pathologies such as Alzheimer’s disease (AD) and Parkinson’disease^25^. The three-dimensional structure of p17, determined by nuclear magnetic resonance and X-ray crystallography, reveals that individual folded p17 molecules are composed of five major α-helixes and a highly basic platform consisting of three β strands^26, 27^. Due to the presence of specific structural motifs defined as “*coiled coil”* sequences, p17 displays a high propensity to misfold and aggregate^28^. This HIV-1 matrix protein is known to possess self-interaction properties since it forms trimers^29, 30^ and hexamers^31^. Two different points of aggregation have been identified in the structure: the C-terminal region (able to interact with the same region of other p17 molecules^32^) and the central matrix portion (aa 42–47)^26^ where the packaging residues are located. The viral protein was found to be expressed in autoptic brain tissue of HIV^+^ patients, mostly confined to the mature macrophages and microglia of fully developed necrotic lesions^33, 34^. Furthermore, p17 has also been detected in the brain tissue of HIV^+^ patients who presented early with severe AIDS encephalopathy^35^. We hypothesised in this study that the ability of p17 to misfold may result in the generation of toxic assemblies in the brain and may be relevant for HAND pathogenesis.

A multidisciplinary integrated approach has been applied to determine the ability of p17 to form soluble amyloidogenic assemblies *in vitro*. To provide new information into the potential pathogenic role of soluble p17 species in HAND, their toxicological potential was evaluated using the invertebrate nematode *Caenorhabditis elegans* as a “biosensor”. This approach is based on knowledge that contraction of the *C*. *elegans* pharynx, fundamental for the worm’s feeding and survival, is inhibited by molecules acting as “chemical stressors”^36^. This *in vivo* model has already been applied to recognise the toxicity of Aβ oligomers and soluble aggregates of amyloidogenic immunoglobulin light chains that play key roles in the pathogenesis of AD and the most common peripheral amyloidosis, respectively^37–40^. We show that p17 significantly inhibits pharyngeal contractions in *C*. *elegans* to an extent comparable to other amyloidogenic proteins, and that its toxic effect is strictly related to its conformational state. Finally, we show that p17 intrahippocampally injected to mice, induced neurocognitive disorders. These findings offer a new way of thinking about the possible cause of neurodegeneration in HIV^+^ patients, which engages the ability of p17 to form soluble toxic assemblies.

## Results

### p17 is present in human brain from HIV-positive patients

**Figure 1:**
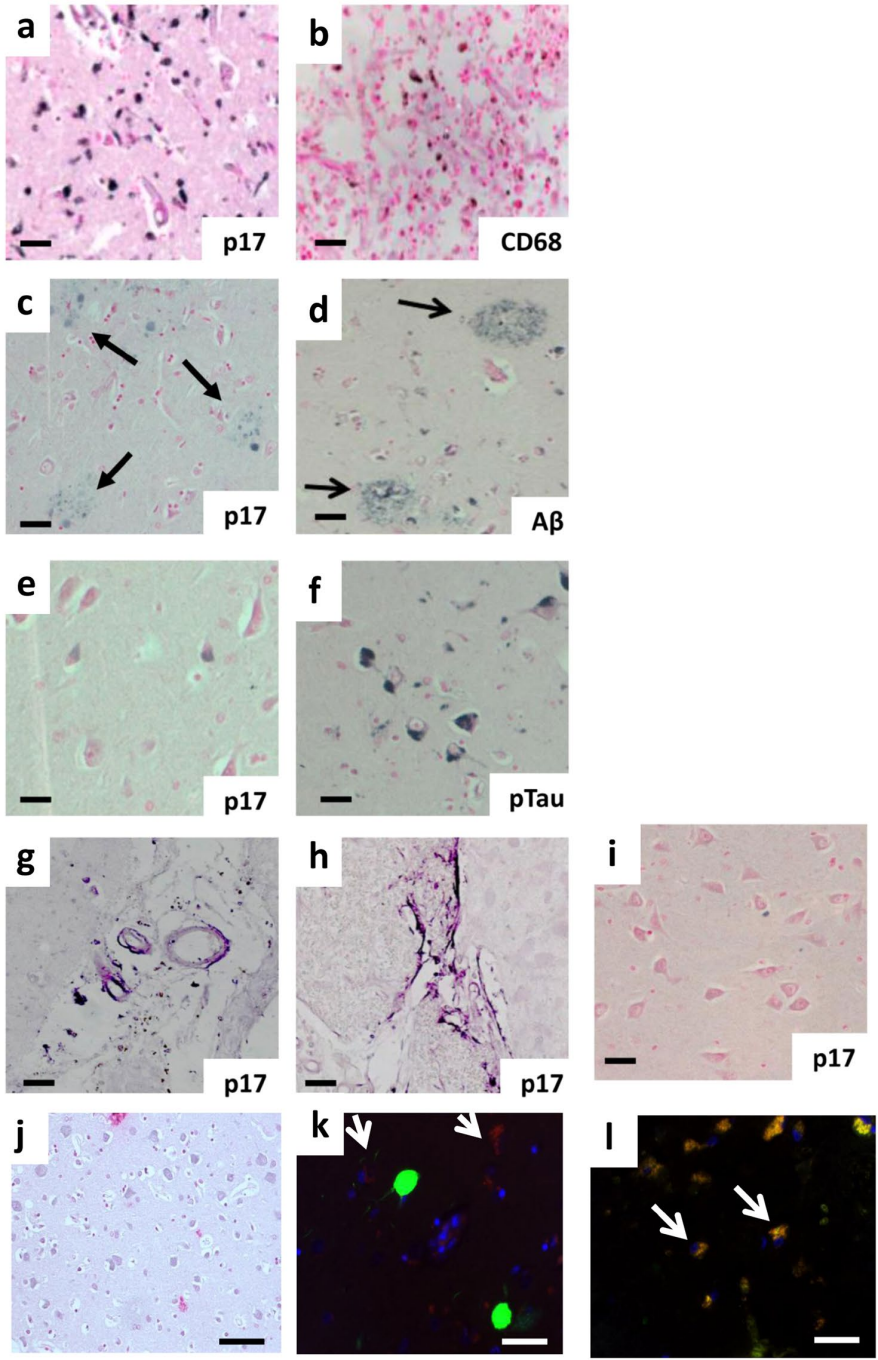
Human HIV-positive brains showed p17-positive staining in inflammatory and neurodegenerative regions. Representative immunohistochemistry of brain sections showing the staining for (**a**) p17 (N-DAB stain, gray-black) and (**b**) CD68-positive macrophages localized to the same region in serial sections as did (DAB stain, brown), (**c**) p17 (N-DAB) and (**d**) β-amyloid (Aβ, DAB stain) positive plaques (black arrows). (**e**) P17-positive (N-DAB) and (**f**) phosphorylated tau (p-tau, DAB stain) positive cortical neurons from the same region. (**g**) Medium sized cortical blood vessels were positive for p17 and (**h**) p17-positive fibril structures within the cortex. (**i**) Shows negative expression of p17 in the cortical region from a non-HIV positive individual and (**j**) a negative control, where the p-17 primary antibody was replaced with PBS during immunohistochemical staining. (**k**) Representative immunofluorescent co-localization of p17 (TRITC-red) and p-tau (FITC-green) and (**l**) β-amyloid (Aβ, FITC-green) in adjacent neurons within the cortex. Nuclei were counter-stained with DAPI (blue). A similar pattern for p17 where yellow/orange demarcates overlapping staining (white arrows) was evident. Representative images are shown from three patient tissue samples used in this immunohistochemical study.(**a**–**j**) Scale bar = 50 µm and (**k**,**l**) = 20 µm.

The presence of p17 in the CNS of patients with AIDS has been sporadically documented in mature macrophages, multinucleated giant cells and in microglia cells^16–18^. In this study the autoptic brains of three HIV-positive patients with HAND were processed and immune-histochemically analyzed to identify the presence of p17 and its localization in specific brain areas. Brain sections from a non-HIV subject which had no history of dementia was analyzed as control. Although the nature of the tissues did not allow to obtain optimal staining patterns, weak p17-positive signals were observed in cortical regions and cortical neurons of HAND’ patients but not within the cortical region of non-HIV-1-infected patients (Fig. 1). The protein was found in the same region with CD68-positive macrophages (Fig. 1a,b), β-amyloid (Aβ) positive plaques (Fig. 1c,d) and phosphorylated tau (p-tau) in serial sections (Fig. 1e and f. Panel k,l shows double immunofluorescence co-regional localization). P17 was also detected in medium sized perforating cortical blood vessels and in many microvessels (Fig. 1g), in accordance with previous observations showing that ECs are a preferential target for p17^25^. Interestingly, fibril structures not associated with cells were also positively stained within the cortex of HIV-positive subjects (Fig. 1h), suggesting that p17 takes part or is *per se* capable of performing fibrillogenesis in the brain parenchyma. Figure 1j shows a negative control section where the p-17 primary antibody was replaced by phosphate buffered saline (PBS). These findings demonstrate that p17 is present in neurodegenerative regions of the brain of HAND’ patients.

### p17 forms soluble oligomeric assemblies

**Figure 2:**
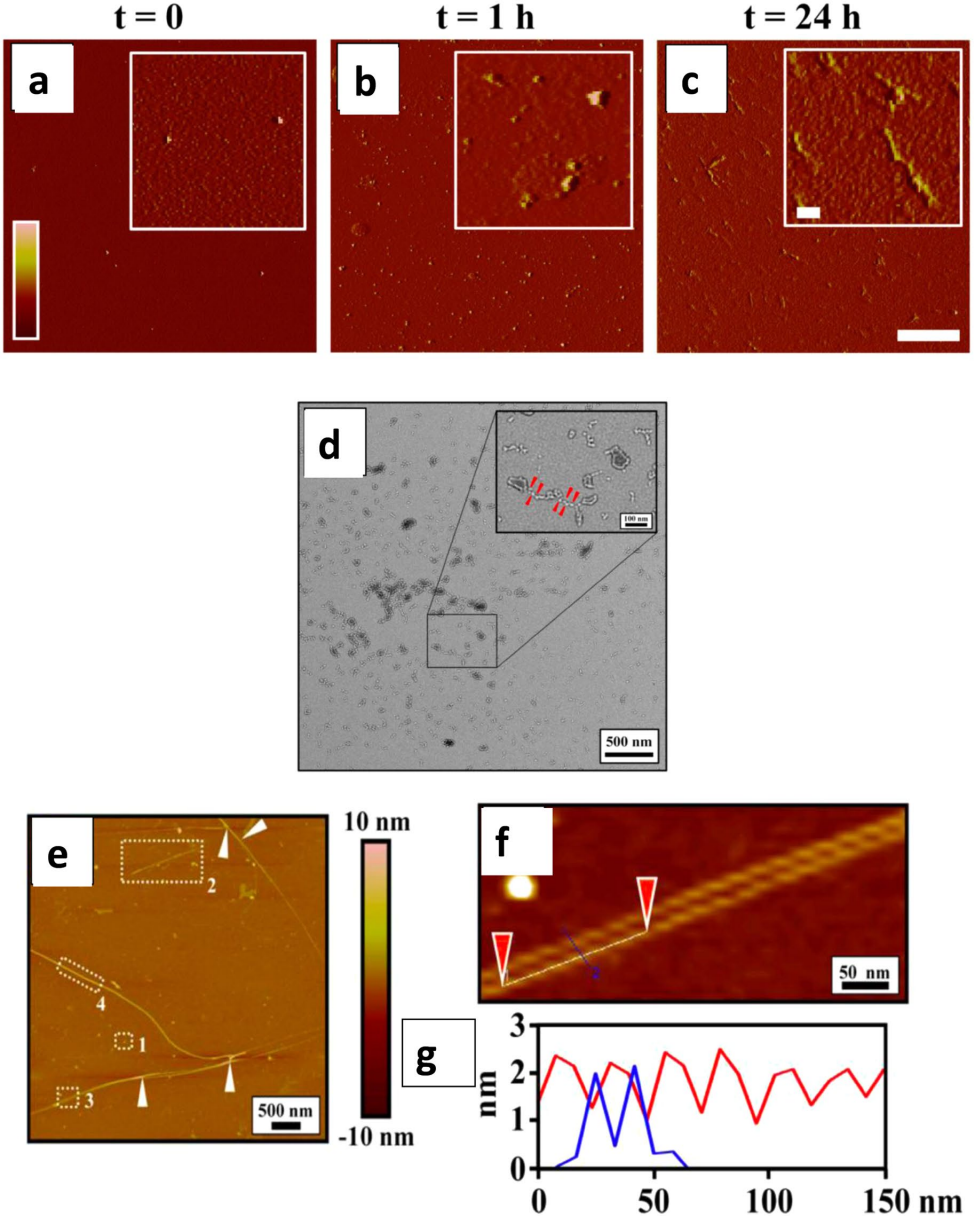
Atomic force microscopy (AFM) and transmission electron microscopy (TEM) analysis of p17. (**a**–**c**) p17 at 4 μM in 10 mM PB (pH 7.4) was incubated at 37 °C for different times (t = 0, t = 1 h and t = 24 h), sampled and left to adsorb on freshly cleaved mica. AFM images were obtained in taping mode and are shown as amplitude data (Z range: −10/+50 mV). Scale bar = 2 µm, inset = 200 nm. (**d**) TEM micrographs of freshly diluted p17 (4 μM in 10 mM PB pH 7.4). The *insets* in *panel* show details of the micrograph at high resolution. The red arrows point to annular structures and rosary-like structures of p17 as an indication of oligomerization process progression. The micrographs are representative of a minimum of 10 different areas. Scale bar = 500 nm, inset = 100 nm. (**e**–**g**) Atomic force microscopy in height of p17 incubated at 37 °C for 24 h shows the presence of globular structures (1), protofibrils (2) and fibrils (3 and 4). The white arrows indicate the presence of twisted protofibrillar subunits. Sample areas marked by dashed rectangles in E3 are reported with a high magnification in (**f**) Moreover, the high profile of fibril analysis, between the positions marked by red arrows, is reported (**g**). (**g**) Sample marked between blue and purple triangles indicate the high profile of a single filament of ~15– 20 nm of diameter and ~2 nm in height.

The ability of p17 to form fibrillar assemblies *in vitro* in cell free condition was then investigated. We previously demonstrated that recombinant p17 maintains a monomeric assembly in high ionic strength solutions, but acquires the propensity to form oligomers when kept at lower ionic strength^41^. To gain better insight into the molecular assemblies of p17 under physiological conditions, we maintained the protein at 37 °C in an isotonic solution. To this end 88 µM monomeric p17 in 0.5 M NaCl was diluted to 4 µM p17 in 10 mM phosphate buffer (PB) (pH 7.4) to obtain a final salt concentration of 0.1 M NaCl. The molecular species that were formed at the different incubation times were characterised using atomic force microscopy (AFM) and transmission electron microscopy (TEM). In a freshly diluted p17 solution (t = 0), oligomers with a range of highly defined dimensions were observed (Fig. 2a). TEM analysis performed on the same sample showed the presence of annular or rosary-like structures as an indication of the presence of small oligomers (Fig. 2d) and confirmed the AFM evidence. Diameters and heights of the assemblies were accurately analysed using the Scanning Probe Image Processor software (Supplementary Fig. S1), which enables elaboration of the AFM images^42^. The majority of the oligomer population (60%) had a diameter in the range of 7–35 nm and no aggregates were detected. When the same sample was incubated for 1 h at 37 °C, larger oligomers ranging from 20 to 96 nm in diameter were observed (Figs 2b and S1). The assemblies increased during the incubation time (Figs 2c and S1) and after 24 h incubation at 37 °C protein aggregates consisting of dense mesh-works of straight, unbranched fibrils of ~15–20 nm in diameter and several µm in length were observed (Fig. 2e,f). After 24 h of incubation p17 formed globular structures, proto-fibrils and fibrils (Fig. 2). The cross-sectional analysis on the top of a fibril segment showed a helicoidal profile along the longitudinal direction of the fibrils with a single filament of ~15–20 nm of diameter and ~2 nm in height (Fig. 2f,g). Notably, samples of p17 after incubation showed an extremely large heterogeneity as far as their morphology and height distribution are concerned, accounting for the presence of a variety of sub-filament constituents (Fig. 2e).

The same samples of p17 were then used to evaluate the exposure of the hydrophobic residues on the surface of the protein. Two fluorescent probes were used for this purpose: 8-anilinonaphthalene sulphonic acid (ANS) and 4,4′-dianilino-1,1′-binaphthyl-5,5′-disulfonic acid (Bis-ANS)^42^. Significant differences in the intensity of the fluorescence emission of ANS was seen between freshly diluted p17 (t = 0) and the same sample incubated for 24 h at 37 °C (Supplementary Fig. S2). This observation indicated the presence of more hydrophobic residues on the external surface of p17 oligomers in the fresh preparation. Moreover, the presence of soluble oligomeric forms with α-helical or random coil/mixed conformers was evaluated by the Bis-ANS assay. Incubation of p17 for 24 h at 37 °C produced a significantly reduced fluorescent signal compared to the sample analysed at t = 0, indicating the presence of larger oligomeric assemblies (Supplementary Fig. S2). As confirmatory information, circular dichroism (CD) analysis was performed to determine the secondary structure of the oligomers. This technique indicated the absence of significant differences among the p17 solutions incubated or not at 37 °C for different times (Supplementary Fig. S3). In HIV-positive subjects circulating p17 is myristoylated. The effect of myristoyl moiety on the secondary structure of myr-p17 was considered and no changes were observed (Supplementary Fig. S3). Both p17 and myr-p17 proteins showed a predominant random-coil conformation with a negative peak in the signal around 210 nm (Supplementary Fig. S3).

### Oligomeric p17 is recognised as “toxic” by *C*. *elegans*

**Figure 3:**
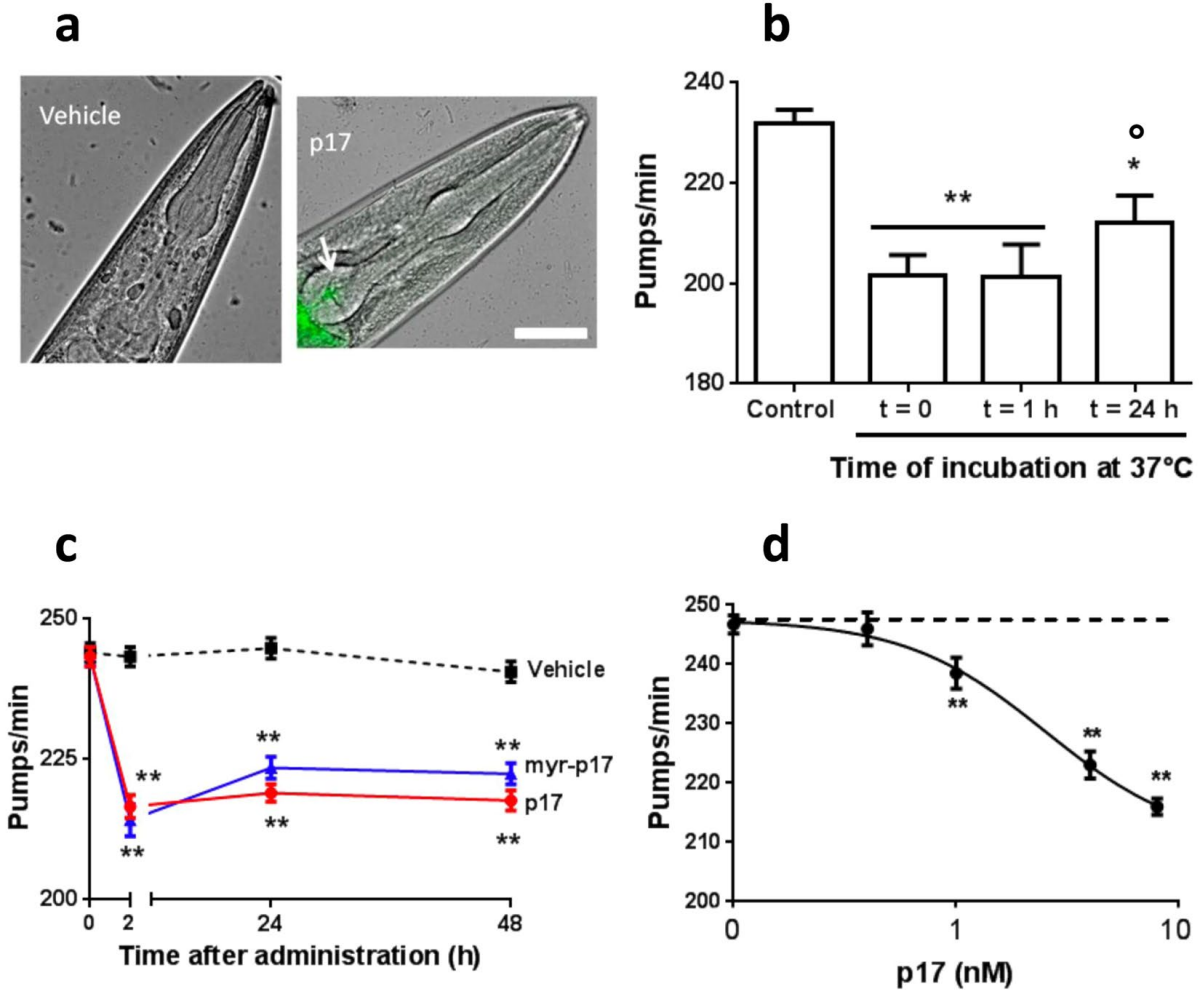
Effect of p17 on *C*. *elegans* pharyngeal motility. (**a**) Representative images of the p17 localisation as overlays of Atto-fluorescence and light microscopy in worms. Scale bar = 50 µm. Worms fed for 2 h vehicle (10 mM PB, pH 7.4) or 4 nM p17-Atto. (**b**) p17 (4 μM in 10 mM PB, pH 7.4) was incubated at 37 °C. At different times (t = 0, t = 1 h and t = 24 h) aliquots were taken, diluted to 4 nM with PB (pH 7.4), and administered to worms. Control worms fed PB (pH 7.4) incubated for 24 h at 37 °C. Pharyngeal pumping was scored by counting the number of pharyngeal contractions in one minute (pumps/minute). *P < 0.05 and **P < 0.01 *vs*. Control, °*P* < 0.05 *vs.* Time 0 and 1 h, one-way ANOVA and Bonferroni *post hoc* test. (**c**) p17 and myr-p17 (at 4 μM in 10 mM PB, pH 7.4) were diluted to 4 nM with PB (pH 7.4), and administered to nematodes as described above. The pharyngeal pumping was scored before plating (t = 0) and at different times after plating. Control worms received vehicle alone (Vehicle). (**d**) Dose-response effect of 0.4–8 nM of p17. Control worms received vehicle alone (Vehicle, dotted line). Data are mean ± SE (N = 30 worms/group). **P < 0.01 *vs*. Vehicle, one-way ANOVA and Bonferroni *post hoc* test.

We investigated the ability of p17 assemblies to exert a toxic effect *in vivo*. First we used the invertebrate nematode *C*. *elegans* which is a well known and established “biosensor” for the rapid recognition of potential toxicity of different amyloidogenic proteins^37–39^. To investigate whether or not the different assemblies of p17 might exert a biological effect on *C*. *elegans*, monomeric p17 (88 µM in 0.5 M NaCl) was diluted to 4 µM in 10 mM PB (pH 7.4), incubated for 1 h or 24 h at 37 °C and administered to the worms at a final concentration of 4 nM, which falls within the range of p17 concentrations detected in the blood of HIV^+^ patients^11^. Under these experimental conditions p17 that had been covalently labelled with the fluorescent dye Atto488 localized to the pharynx of *C*. *elegans* (Fig. 3a). As shown in Fig. 3b, administering freshly diluted p17 to worms (t = 0) caused a significant reduction in the pharyngeal pumping rate (13% with respect to control), and a superimposable effect was observed when worms were treated with p17 pre-incubated for 1 h at 37 °C (t = 1 h). When worms were fed with p17 pre-incubated for 24 h at 37 °C (t = 24 h), an 8% reduction in the pumping rate was observed. These findings indicate that the “toxic” effect of p17 on the pharynx of *C*. *elegans* is related to the folding state of the protein; oligomeric assemblies were specifically recognised in p17 solutions at t = 0 and t = 1 h. The small pharyngeal impairment in worms fed p17 pre-incubated for 24 h at 37 °C might be related to the decrease in hydrophobic residues on the surface of p17. The relationship between protein toxicity and folding was further proven by the absence of effects when worms were exposed to refp17 insAA 117–118 in which the insert destabilised the protein maintaining it in an unfolded state^43^ (Supplementary Fig. S4).

The use of a freshly diluted p17 solution was then chosen to perform all the experiments. To better characterise the temporal ability of the protein to damage the pharynx of *C*. *elegans*, recombinant p17 or synthetic myr-p17^44^ was diluted to 4 nM in 10 mM PB (pH 7.4) and administered to worms for 2 h. The pharyngeal pumping was scored at different times after plating the worms on NGM plates seeded with bacteria. After 2 h the pumping rate of myr-p17-fed worms was significantly reduced compared to vehicle-fed worms (243.3 ± 0.9 and 216.6 ± 2.1 pumps/min for vehicle- and myr-p17-fed worms, respectively) and a comparable inhibition was observed in worms treated with p17 (214.2 ± 1.9 pumps/min) (Fig. 3c). The effects caused by p17 and myr-p17 were comparable to that in worms treated with 10 mM hydrogen peroxide (H_2_O_2_) or 3 µM oligomeric Aβ_1–42_ (Supplementary Fig. S5) here used as positive controls^39, 40^, indicating that the reduction of pharyngeal contraction was biologically important and can translate into a biological phenotype. As expected, the non-amyloidogenic protein purified from the urine of a patient with multiple myeloma, and used as negative control^38^, had no effect (Supplementary Fig. S5). Similar pharyngeal impairments were observed 24 h and 48 h after administering p17 or myr-p17 (Fig. 3c), indicating that both proteins caused permanent, functional damage to the pharynx of *C*. *elegans*. Further experiments have shown that the effects of p17 on worms is dose-dependent and a significant inhibition of pharyngeal pumping was observed at a dose as low as 1 nM p17 (Fig. 3d). The maximum effect was obtained at 8 nM. An IC_50_ value of 2.5 ± 2.0 nM was calculated.

### The 30–55 epitope of p17 plays a key role in the protein’s toxicity

**Figure 4:**
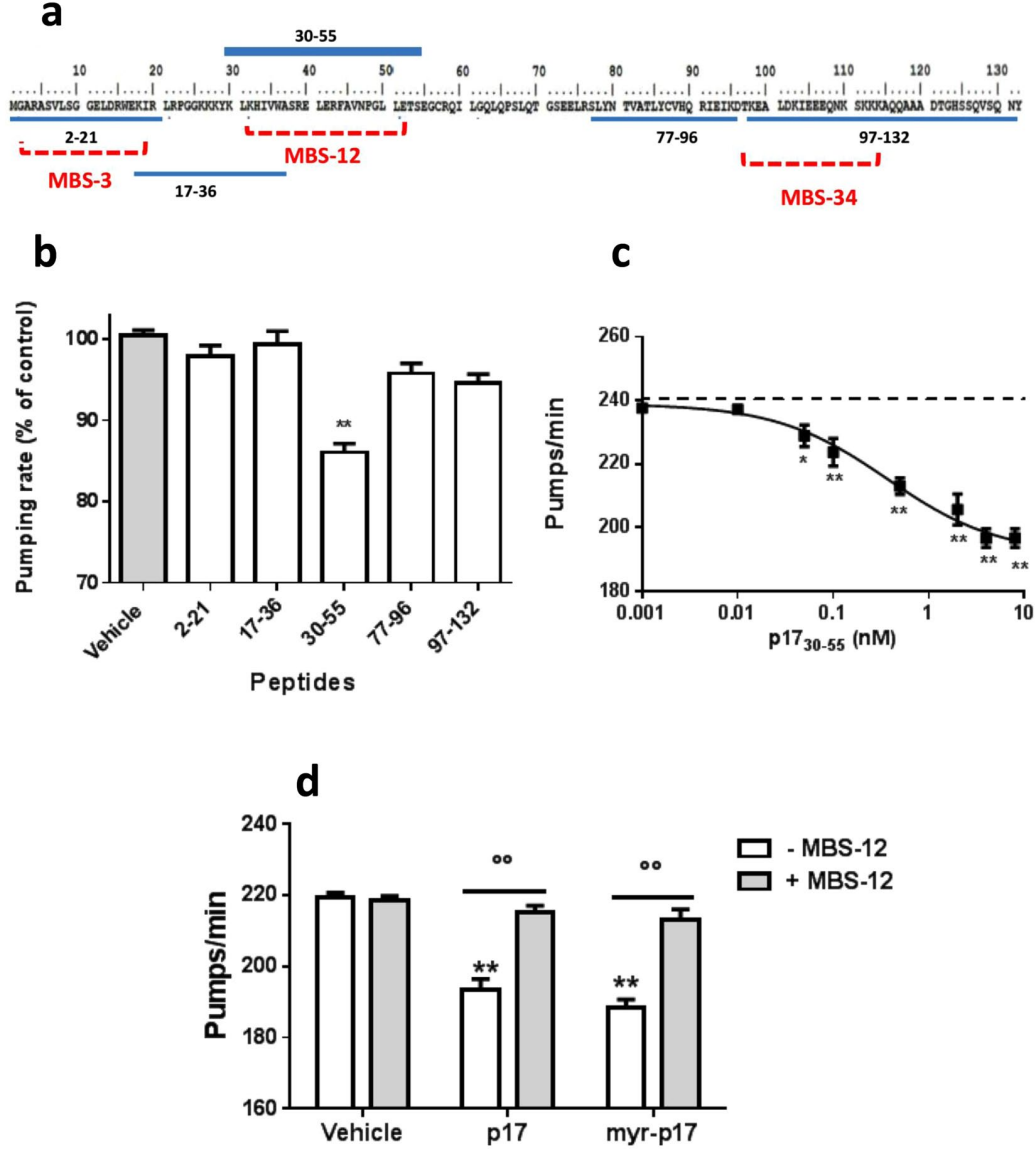
Effect of different epitopes of p17 on its toxicity. (**a**) Amino acid sequence of p17 and of peptides homologous to different residues (blue). In red the epitopes recognised by the different antibodies. (**b**) Effect of different p17 peptides on pharyngeal pumping. Nematodes (100 worms/100 µL) were incubated for 2 h with 4 nM peptides. Control worms received 10 mM PB (pH 7.4) (vehicle). Mean ± SE, **P < 0.01 *vs*. Vehicle, one-way ANOVA and Bonferroni *post ho*c test. (**c**) Dose-response effect of 0.01–8 nM p17_30–55_ peptide. Control worms received vehicle alone (vehicle, dotted line). (**d**) MBS-12 antibody at 6 ng/µL in 10 mM PB (pH 7.4) was co-incubated for 30 min with 4 nM p17 or myr-p17 before administering to the worms. MBS-12 (6 ng/µL) and 10 mM PB (pH 7.4) alone were used as positive and negative controls, respectively (vehicle). Mean ± SE (n = 20 worms/group). **P*p* < 0.01 *vs*. vehicle and °°*P* < 0.01 *vs*. the corresponding p17 or myr-p17, two-way ANOVA and Bonferroni *post hoc* test.

To investigate the role of different epitopes of p17 in pharyngeal toxicity, peptides homologous to different regions of the protein were synthesised (Fig. 4a) and administered to *C*. *elegans*. The effect of a 20 aa-long p17-based sequence (p17_2–21_) homologous to the N-terminal region, known to bind with the chemokine receptors CXCR1 and CXCR2^13, 20^, was first considered. This region is particularly relevant for the detrimental functions of p17 on the immune system, and represents the binding region of the MBS-3 monoclonal antibody directed against AT20 that neutralises the biological activities of the viral protein. Peptides homologous to residues 17–36, 30–55, 77–96 and 97–132 were also evaluated. When administered to *C*. *elegans* at a concentration of 4 nM, only the p17_30–55_ peptide was significantly effective in reducing the pharyngeal pumping of control worms (233.7 ± 2.5 pumps/min) compared to an equimolar concentration of p17 (199.5 ± 2.0 and 206.0 ± 3 pumps/min for p17_30–55_ and p17 treated worms, respectively) (Fig. 4b). No significant effect was observed when worms were treated with 4 nM of a p17_30–55_ scrambled peptide, synthesized as control (231.8 ± 2.5 pumps/min). Both p17_30–55_ and p17_30–55_ scrambled (p17sc_30–55_) peptides were unfolded, as indicated by CD and AFM analysis (Supplementary Fig. S6), suggesting that the toxicity of this aminoacid sequence of the p17 was not necessarily related to its ability to acquire a β-sheet structure.

The IC_50_ value for p17_30–55_ was calculated approximately 7-fold lower than that of p17 (IC_50_: 0.37 ± 1.1 nM and 2.5 ± 2.0 nM for p17_30–55_ and p17, respectively; *p* < 0.01 Student’s *t* test) (Fig. 4c). The relevance of the 30–55 epitope—residing in the second α-helix loop of p17—in driving the pharyngeal toxicity was confirmed by the experiments performed using the MBS-12 antibody, which recognises the amino acids 33–50 in the p17 sequence (Fig. 4a). This antibody–but not MBS-3 (recognising aa 11–18), MBS-34 (recognising aa 97–115), or a p17-unrelated antibody used as negative control–effectively counteracted the pharyngeal dysfunction caused by both unmyristoylated and myristoylated p17 (Figs 4d and S7).

### p17 induces neurocognitive disorders in mice

To further substantiate the observations made in *C*. *elegans*, selected experiments were performed in mice to evaluate the effect of p17 on general behavior and cognitive function. To this end, mice were intrahippocampally injected with 1 µL of p17 (0.5 µg/µL in artificial Cerebrospinal fluid (aCSF)) or aCSF alone (used as control) and different non-cognitive and cognitive behavioral tests were performed at 2-3 weeks after the treatment. A separate experiment was done to discard any potential, non-specific effect of intrahippocampal delivery of exogenous proteins. An additional groups of mice was given 1 µL of 0.5 µg/µL of refp17 insAA 117–118, which had no effect in *C*. *elegans* experiments, or Aβ_35-25_ peptide, used as negative control in the experiments evaluating the neurotoxic effects of peptide Aβ_25-35_ (Supplementary Figs S8 and S9).

**Figure 5:**
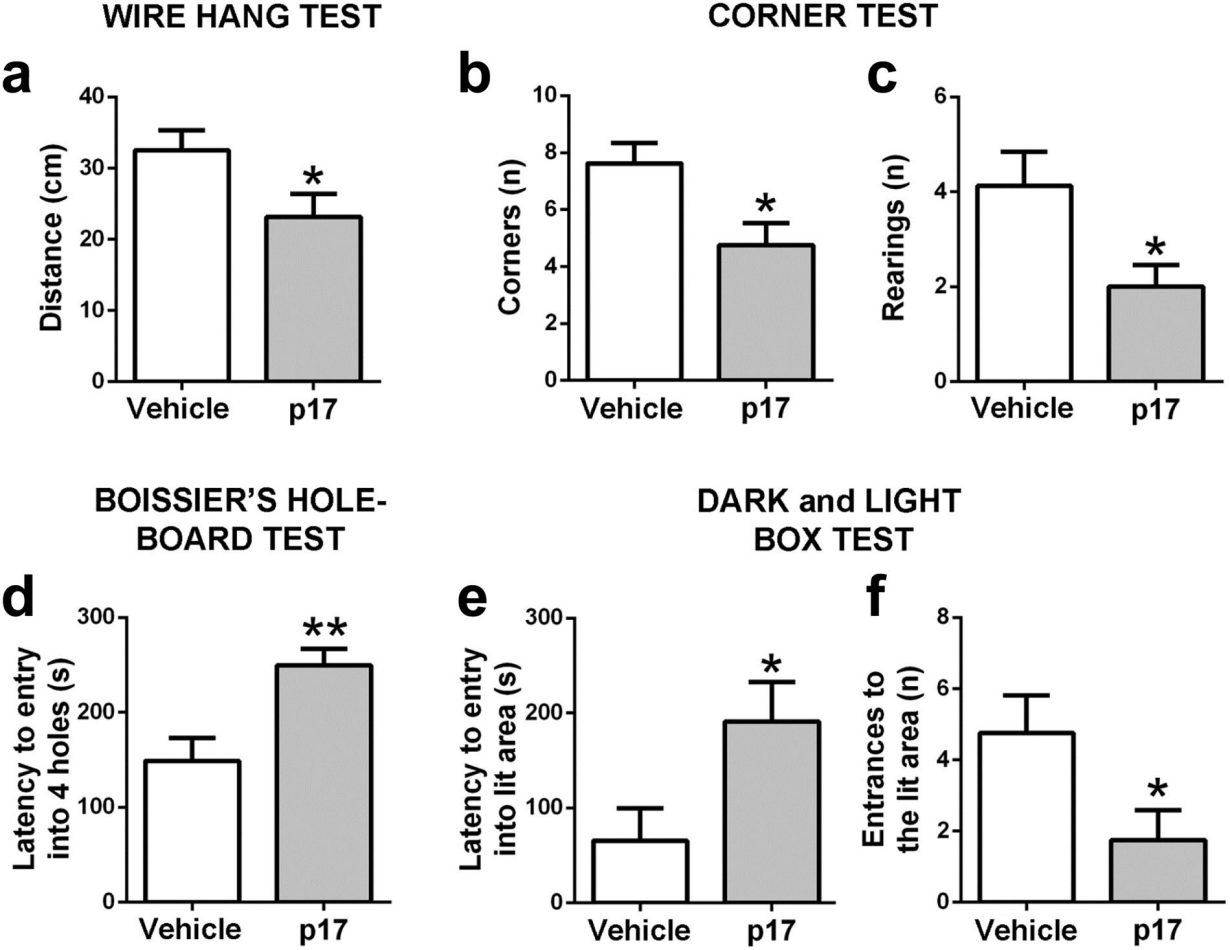
p17-injected mice showed significant deterioration of behavior compared to control mice. (**a**) p17-injected mice (p17) showed lower fine motor coordination as they travelled a significantly shorter distance in the wire hang test in respect to control animals injected with vehicle (Vehicle). (**b**–**f**) p17-injected mice showed behavioral and psychological symptoms of dementia–like, namely neophobia, indicated by (**b**) lower corner explorations and (**c**) lower number of rearings, (**d**) lower exploratory curiosity with higher latency to explore the whole 4 holes in the Boissier’s hole-board test, and (**e**) higher anxiety indicated by increased latency to enter to the lit compartment and (**f**) lower number of explorations in the dark and light box test, than Vehicle animals. Mean ± SE (n = 8 mice/group). *P < 0.05 and **P < 0.01 *vs*. Vehicle, unpaired Student’s t-test.

In the sensorimotor test battery, p17 reduced the fine coordination required to move sideways on the wire during the wire hang test (Fig. 5a). However, reflex responses or coarse motor coordination on a rod were not affected by p17, neither was grip strength when clung on a wire (data not shown). No substantial changes of general activity and behavior were observed in the open field (data not shown) either. Specific tests were performed to detect changes associated to behavioral and psychological symptoms of dementia (BPSD)-like. In this regard, p17 caused a reduced number of corners (Fig. 5b) and rearings (Fig. 5c) when visiting in a new home cage indicative of neophobia, and an increased latency to explore the four holes in the Boissier’s whole-board test (Fig. 5d), indicative of impaired exploratory behavior. Furthermore, p17-treated mice showed increased latency and reduced number of entrances into the lit area of the dark and light box test indicative of anxiety in a stressful environment (Fig. 5e,f). No depression-like behavior as determined in the tail suspension test, was induced by p17 administration (data not shown).

**Figure 6:**
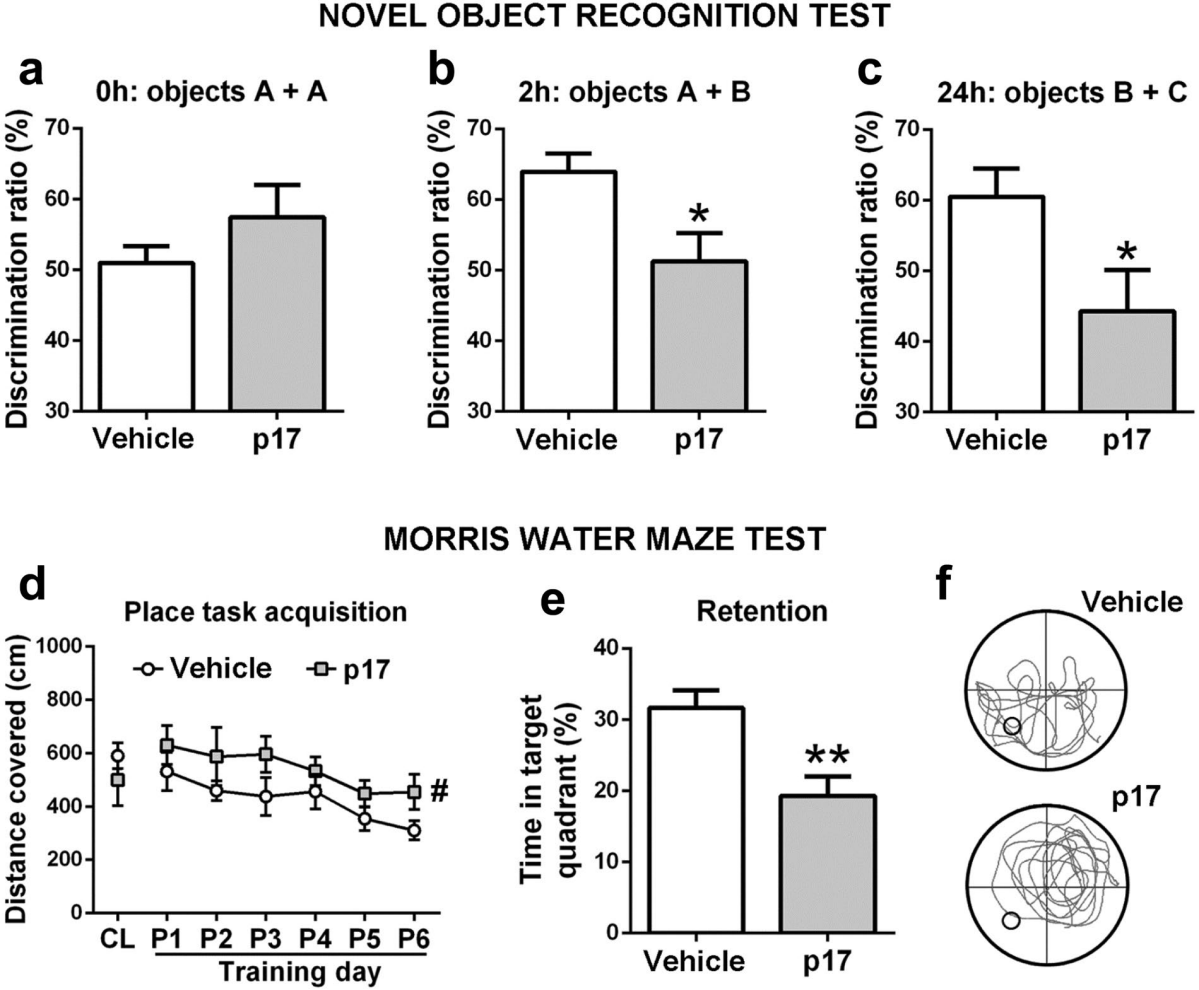
Learning and memory impairment in p17-injected mice. Cognition was tested with the novel object recognition test (NOR) and Morris water maze test (MWM). (**a**–**c**) In the NOR, p17-injected animals (p17) lost the ability to discriminate a novel object from a familial one. Vehicle = Control animals injected with vehicle. (**a**) p17 mice explored similarly to Vehicle mice one of two identical “A” objects in “A + A” at time 0 h, despite higher variability in the former mice. (**b**) p17 mice showed lesser exploration of a novel object “B” than Vehicle mice when exposed to objects “A + B” 2 h later. (**c**) p17 mice also spent less exploration time in a novel object “C” than Vehicle mice when exposed to objects “B + C” 24 h later. (**d**–**f**) In the MWM, p17-injected animals had deficiencies in spatial learning and memory. (**d**) p17 mice showed lower abilities than Vehicle mice throughout six days of training to find the position of a hidden scape platform by relaying on distinctive landmarks around a circular pool (CL = cue learning at day 0, P = place task learning at days 1–6). (**e**) p17 mice spent less time swimming in the pool quadrant where the platform had been located than Vehicle mice when the platform was removed for the probe trial of retention. (**f**) The computer generated tracks showed that p17 mice swam at random, whilst Vehicle mice swam preferentially in the platform quadrant (representative tracks of Vehicle and p17 mice; circle indicates the previous platform position) during the retention trial. Mean ± SE, n = 7 mice/group in (**a–c**) and n = 8 mice/group in (**d**,**e**). Chance performance is 50% in (**a-c**) and 25% in (**e**). *P < 0.05 and **P < 0.01 *vs*. Vehicle, unpaired Student’s t-test in (**b**,**c**,**e**), ^#^ *P* < 0.05 effect of factor treatment in two-way repeated measures ANOVA in (**d**).

The intrahippocampal injection of p17 induced cognitive loss, as detected in two validated tests of learning and memory: the novel object recognition (NOR) and the Morris water maze (MWM). In the NOR, mice administered with p17 showed a similar attention to two identical objects at time 0 of the test procedure, as did control mice (Fig. 6a). As expected, after 2 h and 24 h, the attention time to a novel object increased in control mice compared to time 0 (Fig. 6b,c) as indication of their ability to discriminate a new object versus a familial one. This is not true for p17-treated mice which showed lack of object discrimination memory (Fig. 6b,c).

Reduced acquisition of spatial learning was then demonstrated in the MWM (Fig. 6d). In this test, p17 induced a lower reduction of the distance covered along the 6 days of place task acquisition in respect to controls. Moreover, p17 caused a defect in remembering the quadrant where was previously located the platform (Fig. 6e). These mice demonstrated a search of the scape platform at random throughout the four pool quadrants, whereas control mice showed a preference for the platform quadrant (Fig. 6e). Swimming traces confirmed the random swimming of mice treated with p17 and the preference for the platform quadrant of control mice (Fig. 6f).

**Figure 7:**
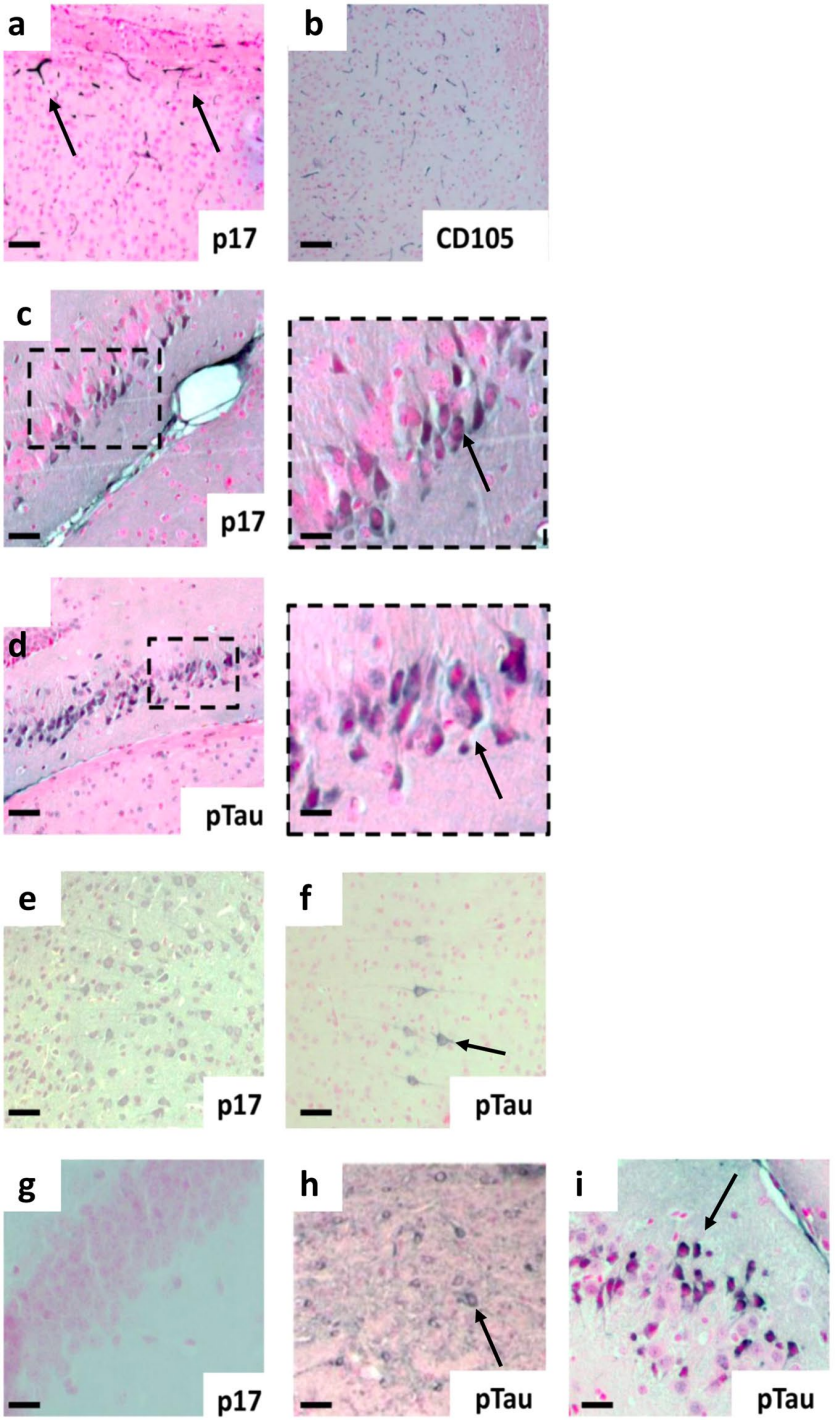
Histological localization of p17 in the mouse hippocampus and local cortical regions. Representative immunohistochemistry of brain sections showing the staining for (**a**) p17 (N-DAB stain, gray-black) into the CA1 region localized to cortical microvessels and (**b**) CD105 (DAB)-positively stained vessels in the same location in a serial section. (**c**) P17 was observed in hippocampal neurons adjacent to the injection point (inset scale bar = 10 µm), (**d**) in the same region as phosphorylated tau (p-tau, DAB) (inset scale bar = 10 µm). (**e**) P17 was found to be expressed consistently within cortical neurons, some of which (**f**) were also p-tau-positive. (**g**) No staining was observed in none-injected controls. (**h**,**i**) P-tau positivity in sham-injected 3xTg mouse. Scale bars (**a**–**h**) = 50 µm, (**i**) = 10 µm. Images show representative staining results from one or more of six animals in each grouping where sectioning throughout the whole bregma was carried out. See Supplementary Information for immunofluorecence and double immunohistochemistry staining patterns as well as primary antibody-negative controls.

**Figure 8:**
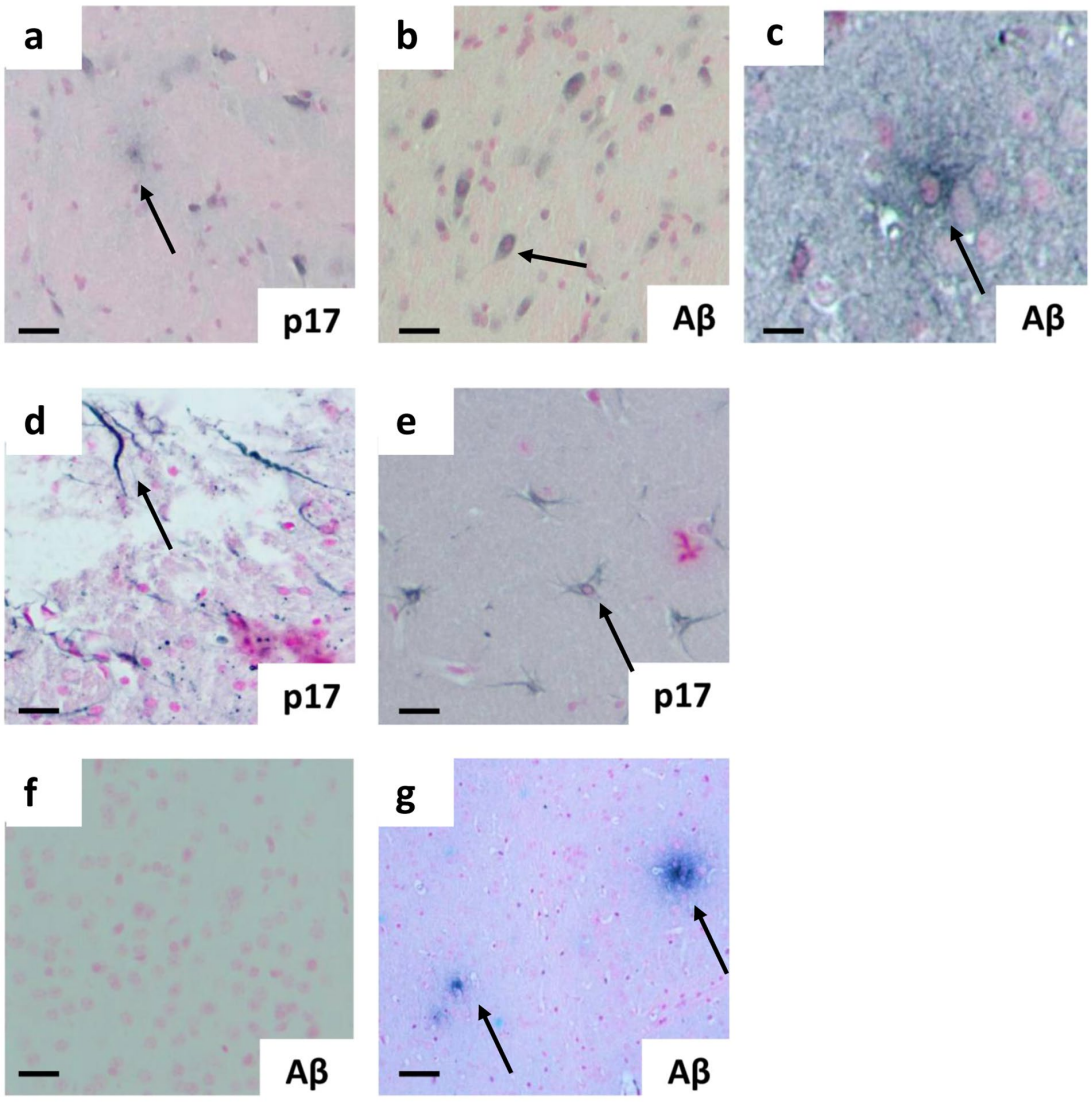
p17 staining localizes to plaque and fibril-like cortical structures in p17-injected mice. Representative immunohistochemistry of brain sections showing the staining for (**a**) p17 (N-DAB stain, gray-black), (**b**) β-amyloid (Aβ, DAB stain) and (**c**) focus on a Aβ plaque in p17-injected cortical regions. (**d**) Fibril-like structures composed of p17 within the local cortex to injection site and (**e**) p17-positively stained astrocytes. (**f**) Lack of Aβ staining in a sham control-injected wild -type mouse and (**g**) positive staining of Aβ was observed in sham-injected 3xTg-positive mice. (**a**–**g**) Scale bar = 50 µm apart from (**c**) = 10 µm. Images show representative staining results from one or more of six animals in each grouping where sectioning throughout the whole bregma was carried out. See Supplementary Information for immunofluorecence and double immunohistochemistry staining patterns as well as primary antibody-negative controls.

To evaluate whether these behavioral defects can be associated to the localization of p17 in specific brain areas, at 1 month after p17 administration animals were examined by histology and immunohistological staining. Positive-p17 staining was observed in hippocampus and surrounding cortical microvessels of intrahippocampal-injected animals (Fig. 7a) but not in control animals (Fig. 7g). P17 was found to be expressed consistently within hippocampal neurons, in the same region as p-tau positive (Fig. 7c,d). Interestingly, using serial sections, we showed that p17 localized near to CD105 positive cortical microvessels (a marker of endothelial cell activation and propensity to angiogenesis) (Fig. 7b). P17 was also found in the ventricular tracts areas of surrounding cortical neurons (Fig. 7e), especially in layers 5 and 6 and more distant cortical areas (primary and secondary motor areas), layers 2–5, some of which were p-tau-positively stained (Fig. 7f). Moreover, as shown in Fig. 8, p17 localized to plaque and fibril-like cortical structures in p17-injected mice and in addition, stimulated β-amyloid expression and plaque-like development within the cortex. In addition, p17 co-localized with p-tau positive cortical and hippocampal neurons and CD105-positive cortical microvessels as well as Aβ-positive cellular regions (Supplementary Figs S10 and S11). These results were also confirmed by the images obtained after immunohistochemistry analysis (Supplementary Figs S10 and S11).

These findings indicate that the cerebral localization of p17 in p17-injected mice was comparable to that seen in cortical regions of HIV-1-infected subject suffering from HAND (Fig. 1) thus enforcing the hypothesis of a key role for p17 as a strong promoter of neurodegeneration.

### Tetracyclines counteract the toxicity of p17

To explore pharmacological approaches aimed at neutralising the toxic activity of p17, a nematode-based approach was employed. The use of *C*. *elegans* for explorative pharmacological studies offers many advantages; it is more rapid, less expensive and avoids the ethical issues that are involved with the use of vertebrate animals.

**Figure 9:**
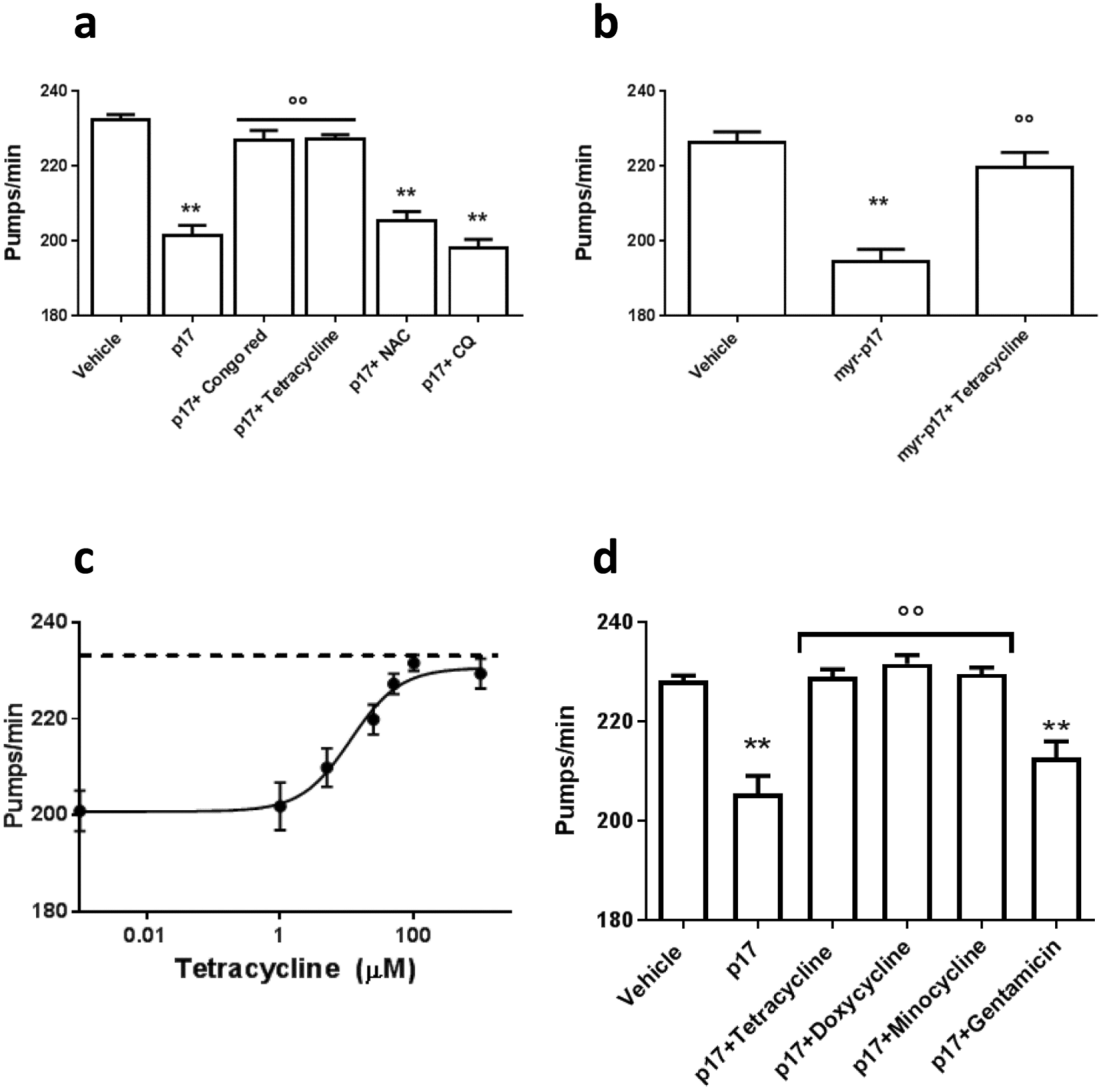
Tetracyclines protect *C*. *elegans* from the toxicity induced by p17. (**a**,**b**,**d**) N2 nematodes (100 worms/100 µL) were incubated for 2 h with 4 nM p17 or myr-p17 in the absence or presence of: 200 µM Congo red, 50 µM tetracyclines, 5 mM *N*-acetylcysteine (NAC), 25 µM clioquinol (CQ), or 50 µM gentamicin, in the absence of OP50 *E*. *coli*. Control worms were fed 10 mM PB (pH 7.4) (vehicle). Mean ± SE (N = 20 worms/group). **P < 0.01 *vs*. vehicle and °°*P* < 0.01 *vs*. p17 alone, one-way ANOVA and Bonferroni *post hoc* test. (**c**) Dose-response effect of tetracycline. Control worms received vehicle alone (dotted line). Mean ± SE (n = 20 worms/group).

The effect of Congo red (a prototypic anti-aggregating compound) and tetracycline hydrochloride (an antibiotic with anti-amyloidogenic activity^38, 45^) in counteracting the p17-induced toxicity was evaluated. We simultaneously considered whether reactive oxygen species and metal ions—known to interact with different amyloidogenic proteins promoting misfolding and generating toxic assemblies^40, 46^ —could be actively involved in p17 toxicity. To this end the effects of a classical antioxidant *N*-acetylcysteine (NAC, 5 mM)^38^ and the metal binding compound 5-chloro-7-iodo-quinolin-8-ol (clioquinol, CQ, 25 μM)^46^ were examined. Congo red at 200 μM and tetracycline at 50 μM completely abolished the inhibition of pharyngeal dysfunction caused by 4 nM p17, whereas no protective effect was observed with NAC and CQ (Fig. 9a). Drugs alone did not affect the pharyngeal pumping rate (Supplementary Fig. S12). NAC and CQ proved to be toxic when administered at higher doses (data not shown). Tetracycline at 50 µM was also effective in counteracting the toxicity induced by myr-p17 (201 ± 1.42 pumps/min and 228 ± 1.31 pumps/min for nematodes fed myr-p17 and myr-p17 + tetracycline, respectively; *p* < 0.001, one-way ANOVA and Bonferroni *post hoc* test; N = 30) and restoring the basal pharyngeal function (232 ± 1.42 pumps/min) (Fig. 9b). The ability of tetracycline to neutralise the pumping dysfunction caused by p17 was dose dependent, with an IC_50_ value of 11.4 ± 0.11 μM and a maximum effect at a dose of 25 μM (Fig. 9c). This effect was unrelated to its antibiotic activity, as indicated by the lack of protection by gentamicin, a drug exerting antibiotic activity only (Fig. 9d). Similar effects were observed for worms fed with doxycycline and minocycline (Fig. 9d), which exhibit a great structural similarity with tetracycline.

To reproduce conditions that are most likely encountered in the clinic, we designed experiments in which tetracycline was administered to *C*. *elegans* when the pharynx was already damaged by p17. Worms were first fed with 4 nM p17 for 2 h before administering tetracycline (50 µM) for 30 min. The drug was capable of repairing the pharyngeal dysfunction caused by p17 (Supplementary Fig. S13), suggesting a potential application for therapeutic use.

To gain insight into the molecular and supramolecular mechanisms underlying the protective effect of tetracycline, we carried out a series of experiments using AFM, TEM and Nuclear Magnetic Resonance (NMR) spectroscopy. The co-incubation of p17 with tetracycline (1:2 molar ratio) for 48 h markedly changed the aggregation state of the protein, as shown by AFM and TEM studies (Supplementary Fig. S14). This data supports the hypothesis of a supramolecular interaction between p17 and tetracycline—similar to that observed between tetracycline and other amyloidogenic proteins^47, 48^— and indicates that this drug may trigger the formation of new, non-toxic protein assemblies. Saturation transfer difference (STD)-NMR spectroscopy^47, 48^ was applied to characterise the molecular recognition events involving p17 and tetracycline. The ^1^H-NMR spectra showed a decrease in the signal intensities over time, particularly for protein signals (Supplementary Figs S14 and S16). This result suggests that p17 can form assemblies of increasing dimensions that cause a broadening, and consequently a decrease, of signal intensities. STD-NMR spectra strongly support the formation of larger p17 assemblies interacting with the drug (Supplementary Figs S15 and S16). The presence of tetracycline signals in the STD spectra, recorded after selective irradiation of the protein resonances offers good proof of interaction between the viral protein and tetracycline and the higher intensity of aromatic resonance suggests that the aromatic ring of tetracycline is strongly involved in this interaction.

Tetracycline exerts its protective activity by increasing the sensitivity of amyloidogenic proteins to protease digestion^49^. To investigate whether tetracycline can affect the sensitivity of p17 to enzymatic degradation, p17 was incubated for 30 min at 37 °C with trypsin in the presence of increasing drug concentrations (0.25–4.0 µg) (Supplementary Fig. S17). To obtain approximately 50% degradation of p17, a protein-to-trypsin ratio of 1:0.03 (w/w) was used (Supplementary Fig. S17). The data obtained indicate that tetracycline does not facilitate the enzymatic degradation of p17.

## Discussion

Previously data on p17 expression in CNS tissue of patients with AIDS revealed the presence of the viral protein in potentially HIV-1-infected elements, such as mature macrophages and microglia cells^19, 21, 50^. In attempt to reinforce these findings, we studied p17 expression in brain tissues of individuals who died with HIV-1 and signs of HAND showing, for the first time, unexpected pathological features of p17 cell localization. The most striking observation was to highlight the presence of p17 in amyloid-like structures in the brain parenchyma mimicking conventional neurodegenerative protein expression in regions identified with plaques/inflammation and or neuronal cell death. Although in a limited numbers, the brains of HIV^+^ -subjects were of senior patients who did not receive cART. These findings, together with p17 expression in ECs and in cortical neurons, opened the way to further study of the possible involvement of p17 in promoting neuropathological degeneration.

Further studies should confirm and detail these observations in a larger cohort of patients and determine if ‘clearing’ of the protein could form the basis of a potential therapeutic approach to prevent neurodegenerative disease.

The capacity of proteins to convert from their soluble forms into highly ordered prefibrillar and fibrillar aggregates is a mechanism common to amyloidogenic neurodegenerative diseases. Although very heterogeneous from a clinical point of view, all these pathologies are characterised by the ability of an endogenous protein to accumulate in the brain and misfold, assuming toxic molecular assemblies. The ability of the endogenous protein to form oligomers is considered critical for the insurgence of several neurodegenerative diseases^51^. In this study we provide a characterisation of the morphology and spatial extent of the assemblies produced by the HIV-1 matrix protein p17 under physiological conditions. Starting from a monomeric form, recombinant unmyristoylated p17, as well as synthetic myr-p17, changes conformation to become small oligomers that grow rapidly with time and form annular or rosary-like structures before forming prefibrillar soluble structures. Among the various chemico-physical mechanisms involved in the toxicity cascade of amyloidogenic proteins, the exposure of hydrophobic surfaces is one of the early events^52^. We have shown that hydrophobic residues exposed on p17 oligomers and their presence on the external surface of the protein was increased during the aggregation process.

The biological relevance of the assemblies formed by p17 was then investigated using the *C*. *elegans*-based assay already used to recognise the toxic oligomers of soluble amyloidogenic proteins^37–39^. Its validity has been proven by the demonstration that the Aβ oligomers recognized as toxic by *C*. *elegans* also cause cognitive impairment in a mouse model of Aβ-memory dysfunction^53^. Feeding worms with soluble oligomers of unmyristoylated or myr-p17 permanently impaired the pharyngeal function in a dose-dependent manner. Interestingly, the impairment was maximal at concentrations comparable to those commonly found in blood of HIV^+^ patients. The ability of both p17 preparations to exert toxic effects on the worm’s pharynx was closely related to their folded status, since an unfolded p17 protein was completely ineffective. The exposure of hydrophobic residues on the surface of p17 oligomers may be important for the dysfunction caused by the protein on the *C*. *elegans* pharynx, as suggested by the lower effect when worms were fed with larger assemblies instead of small oligomers. All these findings support that the toxic effect of p17 may contribute to the toxicity of p17 in the HIV-infected brains.

Extracellularly, p17 has been found to deregulate the function of different target cells involved in AIDS pathogenesis^11, 22, 54, 55^. All activities of p17 rely on interactions between the AT20 functional domain located at the N-terminal region and specific receptors expressed on different target cells^18^. Recent studies have shown the capability of p17 to exert chemokine^20, 41^, pro-angiogenic^13^ and pro-lymphangiogenic activities^19^ after binding to the chemokine receptors^13, 20^. *C*. *elegans*, similar to other invertebrates, has an ancestral immune system devoid of specialised immune cells and adaptive immunity. This worm recognises and defends itself against viral infections by activating the intracellular RNA interference machinery; it defends itself against bacterial pathogens by activating the MAPK signalling pathways^56^. There is no evidence of the existence of a chemokine system in *C*. *elegans* and chemokine or chemokine receptor-like sequences have not yet been found^57^. This knowledge explains why MBS-3, a p17 neutralising monoclonal antibody directed against AT20^22^ does not neutralise the p17 toxicity. On the contrary, MBS-12 antibody directed against the central region of p17 (aa 30–55), was able to neutralise p17 toxicity, showing that this region takes direct part in mediating the toxic effects of p17 on *C*. *elegans*. The involvement of this region in p17 toxicity was further assessed by using a synthetic peptide mimicking the region spanning residues 30–55. With this peptide the observed toxic effect on the worms was greater than when the entire protein was used.

The p17 toxic epitope for *C*. *elegans* that we have identified constitutes charged and hydrophobic residues that are mostly located on the outside of the protein^26^. It is well known that mutations of critical residues within the region responsible for p17 toxicity leads to unfolded proteins^50^, suggesting that this functional epitope is structurally constrained. In this study we show that an unfolded p17 protein characterised by an Ala-Ala insertion in the p17 C-terminal region^43^ is completely devoid of toxic activity. This finding confirms that the p17-mediated pathogenic effect observed in *C*. *elegans* involves interactions between this newly identified p17 functional region and still unknown cellular proteins, and that such interactions, consequently the p17 toxic effect, can be abolished by gross distortion of p17 structure.

The *in vivo* toxic effect of p17 was confirmed by data generated in mice injected showing that direct injection of the protein into the hippocampus resulted in deficiencies in sensorimotor, behavioral response and cognitive capacities similar to those seen in transgenic mouse models of AD at advances stages of pathology^58, 59^. A reduction of fine sensorimotor abilities indicated initial deterioration of motor coordination present in many advanced stages of cognitive loss and neurodegeneration. Furthermore, the presence of a variety of behavioral disorders, including neophobia, anxiety and apathy, indicates that p17 induced the development of a set of disturbances similar to the behavioral and psychological symptoms of dementia (BPSD) associated to AD. BPSD occur in 50–90% of AD patients and is caused by imbalance of cerebral neurotransmitters in diverse brain areas responsible for emotional activities^60^. Whereas p17 caused a moderate level of BPSD-like alteration, probably because of the hippocampus local injection, it was able to cause a total lack of learning and memory in the MWM and the NOR tests. Spatial learning and memory in the former test is mainly a hippocampus-based task^61^ whereas hippocampus is also an important area for recognition memory in the later^62^. Therefore, the extreme cognitive deterioration observed in mice after the administration of p17 would agree with the brain region of injection.

The hippocampus is an essential brain area in learning and memory mechanisms and it is especially vulnerable to AD-induced degeneration and atrophy^62, 63^. P17 induced hippocampus-related cognitive loss in the NOR and MWM tests indicating that this brain region was very sensitive to the pathological changes induced by this protein. Moreover, p17 mimicked symptoms of AD, being the effects comparable to that observed in AD transgenic mice^63, 64^.

Our data highlight the presence of p17 deposits in the brain parenchyma mimicking conventional neurodegenerative protein expression in regions identified with plaques/inflammation and or neuronal cell death. The characterisation of the morphology and spatial extent of the assemblies produced *in vitro* by the HIV-1 matrix protein p17 indicate that the protein can form small oligomers that grow rapidly with time and form annular or rosary-like structures before forming prefibrillar soluble structures. These assemblies are toxic *in vivo* in *C*. *elegans* and induced neurocognitve disorders in mice.

We showed that tetracycline is effective in counteracting the pharyngeal impairment caused by p17 in *C*. *elegans*. Due to the multiple mechanisms of action^45^, this drug exerts beneficial *in vitro* and *in vivo* effects against a variety of amyloidogenic proteins^45, 48, 49, 65, 66^. Significant hints have come from a study recently performed on patients affected by Creutzfeldt-Jakob disease. These patients received 100 mg doxycycline per day for about 6 months in the absence of side effects^67^. The results indicate that doxycycline can cross the blood–brain barrier and persist in the brain for days to reach concentrations in the low micromolar range^67^. These findings support the potential application of tetracycline or other anti-amyloidogenic compounds already used in human therapy for future translation to clinical use. We are convinced that drug-repositioning strategies are likely to be of particular importance for chronic diseases requiring polytherapy, notably enabling a significant reduction in associated costs.

Our findings offer a new way of thinking about the possible cause of neurodegeneration in HIV^+^ patients, which engages the ability of p17 to form soluble toxic assemblies and put forward innovative hints for the development of novel pharmacological strategies.

## Methods

### Human brain tissue samples

HIV-1^+^ brain samples were obtained from the Brain Bank, Hospital Universitari Mútua de Terrassa, Catalonia. The study was approved by the institutional review board and local ethical committee of the hospital. The tissue samples were collected within 4 h of death from the refrigerated bodies of three HIV-1-infected patients who died from hemorrhagic stroke (male, aged 57, A19–92); ischaemic stroke (female aged 88, A50–96) and aortic aneurysm (male aged 72, A19–97). The diagnosis of probable AD was confirmed by anatomopathology according to standard protocol and Braak classification. Prior to death, all patients signed informed consent. Patients attended an HIV outpatient clinic for at least 2 years and had not received any antiretroviral treatment (this was before cART). On a routine visit, patients were screened for neurocognitive impairment. Cognitive impairment was established when the patient scored above 1 standard deviation below the mean of corrected normative data for age, education and other standards recommended in the standardized neuropsychological assessment in at least two neurocognitive areas. To decide if neurocognitive impairment was HAND, differential diagnosis was carried out following the internationally accepted recommendations. Non-HIV brain tissue was obtained from the Brain Bank (Bristol, UK). The control patients had no history of dementia and sections were specifically from the cortical region, frontal lobe. Immunohistological analysis were carried out on human brains to determine the localization of p17 as described in Supplementary Methods.

### p17 and p17-neutralising antibodies

Purified endotoxin-free recombinant HIV-1 matrix protein p17 (in its monomeric form) and refp17 insAA 117–118 were produced as previously described^43, 68^. To maintain the proteins in their monomeric form, they were stored at −80 °C in concentrations of 80–100 µM in a solution containing 0.5 M NaCl. The absence of endotoxin contamination (<0.25 endotoxin units/mL) in protein preparations was assessed by Limulus amoebocyte assay (Associates of Cape Cod). P17 was covalently labelled with the fluorescent dye Atto488 according to the manufacturer’s instructions (Lightning-Link® Rapid Atto488, Innova Bioscience, Cambridge, UK). The p17 neutralising monoclonal antibodies MBS-3, MBS-34, MBS-12 and an unrelated monoclonal antibody to p24 protein (ap24) were produced as described^69^. Myristoylated HIV-1 matrix p17^44^ (myr-p17) was a kind gift from W. Lu (Institute of Human Virology, University of Maryland Biotechnology Institute, Baltimore, USA).

### *C*. *elegans* studies

Bristol N2 nematodes were obtained from the *Caenorhabditis elegans* Genetic Center (CGC, University of Minnesota, USA) and propagated at 20 °C on solid nematode growth medium (NGM) seeded with *E*. *coli* OP50 (from CGC) for food^38^. To evaluate the effect of conformational changes of p17 on pharyngeal toxicity, p17 was administered to worms (100 worms/100 μL) immediately after dilution at 4 nM in 10 mM PB (pH 7.4) (time 0) and 1 to 24 h after incubation at 37 °C. Control worms were fed with 10 mM PB (pH 7.4) (vehicle). Incubation of the worms with p17 was performed in the absence of *E*. *coli* to avoid any potential interference between bacteria and the protein. Worms (100 worms/100 μL) fed, in the same experimental conditions, 10 mM H_2_O_2_ or 3 µM oligomeric synthetic Aβ_1–42_ prepared as already reported^37^, known to significantly inhibit the pharyngeal pumping rate^38^ and here used as positive controls. As negative control we used a non-amyloidogenic immunoglobulin light chain purified from a patient suffering from myeloma (myeloma, 100 µg/mL) and known to have no effect on pharyngeal activity^38, 40^. After a 2-h incubation with orbital shaking, worms were transferred onto NGM plates seeded with OP50 *E*. *coli*. The pharyngeal pumping rate, measured by counting the number of times the terminal bulb of the pharynx contracted over a 1-minute interval (pumps/min), was scored 2 to 48 h later. Experiments were also performed feeding worms (100 worms/100 μL) with a freshly prepared solution of p17 (0.001–10 nM), myr-p17 (4 nM), refp17 insAA 117–118 (4 nM), p17_30–55_ (0.001–10 nM), or p17_30–55_ scrambled (p17sc_30–55_), p17_2–21_, p17_17–36_, p17_77–96_ and p17_97–132_ all at 4 nM in 10 mM PB (pH 7.4). Control worms were incubated with 10 mM PB (pH 7.4) (vehicle) only. After 2 h of incubation with orbital shaking, worms were transferred onto NGM plates seeded with OP50 *E*. *coli*. The pharyngeal pumping rate, measured by counting the number of times the terminal bulb of the pharynx contracted over a 1-minute interval, was scored 2 to 24 h later.

In selected experiments worms were fed for 2 h with 4 nM p17 or 4 nM myr-p17 alone or with: 5 mM *N*-acetyl cysteine (NAC, Sigma-Aldrich), 25 µM 5-chloro-7-iodo-quinolin-8-ol (clioquinol, CQ, Sigma-Aldrich), 200 µM Congo red (Sigma-Aldrich), 0.1–100 μM tetracycline hydrochloride (tetracycline, Sigma-Aldrich), 50 μM doxycycline (Sigma-Aldrich), 50 μM minocycline (Sigma-Aldrich), 50 μM gentamicin (Sigma-Aldrich). Higher concentrations of NAC or CQ were toxic for the worms. Worms were then transferred onto fresh NGM plates seeded with *E*. *coli* in the presence of the same drug concentrations and the pharyngeal pumping rate was scored after 2 h. Worms were also exposed to the drugs alone or to vehicle in the same experimental conditions.

The effect of antibodies recognising different epitopes of p17 was also investigated. To this end, nematodes were fed for 2 h with 4 nM p17 or myr-p17, pre-incubated or not for 30 min at room temperature with 6 ng/µL of MBS-3, MBS-12, MBS-34 or ap24 antibodies. Nematodes were then transferred to NGM plates seeded with fresh OP50 *E*. *coli* and the pumping rate was scored after 2 h.

### Animals and treatment

C57BL6 male mice were bred from the Spanish colony established in the Medical Psychology Unit, Autonomous University of Barcelona. Animals were individually housed in Macrolon cages (Techniplast, Italy) with free access to food and water and maintained in a temperature controlled room (22 ± 2 °C) with 12 h light/12 h dark cycle. Animal handling, including surgical procedures, behavioral testing and necropsies, was performed at the facilities of the Animal Unit of the University of Barcelona, Spain. The study was approved by the local animal experimentation ethics committee “Comité Ètic d’Experimentació Animal” of the University of Barcelona, under the permit DAAM #6991, issued by the Departament d’Agricultura, Ramaderia, Pesca, Alimentació i Medicine Natural of the Generalitat of Catalonia. All procedures were carried out in accordance with the decree 214/1997 of the Generalitat and the Spanish legislation concerning the protection of animals used for experimental and other scientific purposes and the European Commission Council Directive 86/609/EEC on this subject.

The p17 protein was delivered into the mouse hippocampus by stereotactic surgery procedures. Four-month old mice (n = 16) were anesthetized with xylacine (10 mg/kg, intraperitoneal (i.p.)) (Rompun 2%, Bayer, Leverkusen, Germany) and ketamine (80 mg/kg, i.p.) (Ketolar 50 mg/mL, Pfizer, Alcobendas, Madrid, Spain) and placed in a stereotactic apparatus (David Kopf Instruments, Tujunga, CA). Bilateral infusions of 1 µL of p17 (0.5 µg/µL in artificial cerebrospinal fluid (aCSF)) were performed into the CA1 area of the hippocampus of 8 mice. Control animals (Vehicle, n = 8) were infused with the same volume of aCSF alone (148 mM NaCl, 3 mM KCl, 1 mM CaCl_2_, 0.8 mM MgCl_2_, 0.8 mM Na_2_HPO_4_, 0.2 mM NaH_2_PO_4_). Injections were performed at a rate of 1 μL/min at coordinates relative to bregma of −2.0 mm A/P, ±1.2 mm M/l, −1.5 mm V/D. One µL of the testing solutions was delivered to the application point with a 25-gauge stainless steel cannula (Small Parts Inc., Miami, FL) connected to a Hamilton syringe through a Teflon tube. The syringe was attached to a micro-infusion pump (Bioanalytical systems Inc., West Lafayette, IN). The cannula was left in position for 5 min after delivery to prevent the solution from surging back. Animals were tested for changes of non-cognitive and cognitive behavior at 2-3 weeks after hippocampal injections. A battery of tests were applied on 14 daily consecutive sessions (see Supplementary Methods).

Intracerebral injection into the rodent hippocampus has been widely used by us and other authors and from our experience and published work, no effects on behavior or cognitive deleterious effects are normally associated with the surgery or in the presence of non-active/control proteins^59^.

For a further confirmation, a new set of animals were injected bilaterally in the hippocampus with 1 µL of aCSF (Vehicle, n = 6), 1 µL of refp17 insAA 117–118 at the concentration of 0.5 µg/µL in aCSF (refp17, n = 7), or 1 µL of Aβ_35–25_ (inactive control for Aβ_25-35_; Neosystem, Strasbourg, France) at the concentration of 0.5 µg/µL in aCSF (n = 6). Animals were analyzed for discarding any effect on non-cognitive and cognitive behavior. For immunohistochemistry/ immunofluorescence, the brains of six animals per group were subjected to sectioning throughout the whole bregma and examined every 10^th^ section (see Supplementary Methods).

### Statistical analysis

The data were analysed using GraphPad Prism 6.0 software (CA, USA) by an independent Student’s *t*-test, one-way and two-way ANOVA and Bonferroni’s *post hoc* test analysis. The values of IC_50_ and median survival were determined using Prism version 6.0 for Windows (GraphPad Software, CA, USA). A *p* value < 0.05 was considered statistically significant. At least three independent experiments were done for studies involving *C*. *elegans*. For mouse main experiments, on the basis of advice from a medical statistician and our team’s experience of behavioral studies, 12 were used for each group of mice, Vehicle and p17, providing the minimum number to show up significant data. Column statistics were used in the probe trial of the MWM.

### Human tissue samples

All procedures performed in studies involving human participants were in accordance with the ethical standards of the institutional and/or national research committee and with the 1964 Helsinki declaration and its later amendments or comparable ethical standards. Brain samples were obtained from the Brain Bank, Hospital Universitari Mútua de Terrassa, Catalonia. The study was approved by the institutional review board and local ethical committee of the Hospital Universitari Mútua de Terrassa, Catalonia, Spain.

## Electronic supplementary material

Supplementary Information
