# Supplementary material for: HIV-1 matrix protein p17 misfolding forms toxic amyloidogenic assemblies that induce neurocognitive disorders
Source: Sci Rep. 2017 Sep 4;7:10313. doi: 10.1038/s41598-017-10875-0 (PMC5583282; doi:10.1038/s41598-017-10875-0)
Supplement: Supplementary file 1 — Supplementary Information [file 41598_2017_10875_MOESM1_ESM.pdf]

SUPPLEMENTARTY INFORMATION

HIV-1 matrix protein p17 misfolding forms toxic amyloidogenic assemblies that induce neurocognitive disorders

Yasmin Zeinolabediny [1§], Francesca Caccuri[2§], Laura Colombo[3], Federica Morelli[3], Margherita Romeo[3], Alessandro Rossi[3], Silvia Schiarea[4], Carlotta Ciaramelli[5], Cristina Airoldi[5], Ria Weston[1], Liu Donghui[1], Jerzy Krupinski[1,6], Rubén Corpas[7], Elisa García-Lara[7,8], Sara Sarroca[7], Coral Sanfeliu[7], Mark Slevin[1,8,9], Arnaldo Caruso[2], Mario Salmona[3], Luisa Diomede[3]*

[1]*School of Healthcare Science, John Dalton Building, Manchester Metropolitan University, Chester Street, Manchester, M1 5GD, UK.*
[2]*Department of Molecular and Translational Medicine, University of Brescia, Piazza del Mercato 15, 25121 Brescia, Italy.*
[3]*Department of Molecular Biochemistry and Pharmacology, IRCCS- Istituto di Ricerche Farmacologiche "Mario Negri", Via G. La Masa 19, 20156 Milano, Italy.*
[4]*Department of Environmental Health Sciences, IRCCS- Istituto di Ricerche Farmacologiche "Mario Negri", Via G. La Masa 19, 20156 Milano, Italy.*
[5]*Department of Biotechnologies and Biosciences, University of Milano Bicocca, Piazza dell'Ateneo Nuovo 1, 20126 Milano, Italy.*
[6]*Hospital Universitari Mútua de Terrassa, Department of Neurology, Terrassa, Barcelona, Spain.*
[7]*Institut d'Investigaciones Biomèdiques de Barcelona, CSIC and IDIBAPS, Barcelona, Spain.*
[8]*University of Medicine and Pharmacy, Targu Mures, Romania.*
[9]*Department of Pathology/Medicine, Griffith University, Brisbane, Australia.*

§The authors equally contributed to the work.

*Correspondence:
Email: luisa.diomede@marionegri.it
Tel: +3902390141
Fax: +390239014744

SUPPLEMENTARY METHODS

Synthesis and chemical characterisation of peptides. Peptides homologous to p17 sequence were synthesised: 2–21 ($p17_{2-21}$, $_{H2N-}$GARASVLSGGELDRWEKIRL$_{-COOH}$), 17–36 ($p17_{17-36,}$ $_{H2N-}$EKIRLRPGGKKKYKLKHIVW$_{-COOH}$), 30–55 ($p17_{30-55}$, $_{H3COC}$LKHIVWASRELERFAVNPGLLETSE$_{-CONH2}$), 77–96 ($p17_{77-96,}$ $_{H2N-}$SLYNTVATLYCVHQRIEIKD$_{-COOH}$), 97–132 ($p17_{97-132,}$ $_{H2N-}$TKEALDKIEEEQNKSKKKAQQAAADTGHSSQVSQNY$_{-COOH}$).

A $p17_{30-55}$ scrambled peptide ($p17sc_{30-55,}$ $_{H3COC-}$HLVFEAEWPNGLSKIESLRVATREL$_{-CONH2}$) with a hydrophobic profile comparable to that of $p17_{30-55}$ was synthesised as a control. Peptides were synthesised by solid-phase FMOC chemistry on an Applied Biosystems 433A peptide synthesiser (Life Technologies, Monza, Italy) using L-amino acid derivatives and Rink amide MBHA resin on 0.1 mM scale [1]. Peptides were cleaved from the resin by incubation for 2.5 h with a trifluoroacetic acid (TFA) solution containing 5% phenol, 5% 3,6-dioxa-1,8-octanedithiol, 5% water and 2.5% Triisopropylsilane. The peptides were then purified by reverse-phase HPLC on a semi-preparative Jupiter Proteo 4 µm column (250 x10 mm, 90 Å, Phenomenex, Torrance, CA, USA) on a System Gold HPLC (Beckman Coulter, Milan, Italy) using a water/acetonitrile gradient elution. Identity and purity of the peptides were determined by MALDI-time-of-flight-time-of-flight (TOF/TOF) mass spectrometry (MS) with an ABI 4800 MALDI-TOF/TOF mass spectrometer (Applied Biosystems, Framingham, MA, USA) operating in reflector positive-ion mode. Peptide purity was also checked by reverse-phase HPLC using a System Gold HPLC equipped with an EcoCART 125-3 LiChrospher 60RP-selected B 5 µm analytics column (Merck, Darmstadt, Germany) and UV/VIS detector (214 nm). The purity was always higher than 95%. Peptides were lyophilised and stored at -80°C.

Atomic force microscopy (AFM). p17 and myr-p17 were analysed immediately after their dilution at 4 μM in 10 mM phosphate buffer (PB), pH 7.4 (t = 0) and after a 1 or 24 h incubation at 37°C. $p17_{30-55}$ and $p17sc_{30-55}$ were diluted to a final concentration of 50 μM in 10 mM PB (pH 7.4) and analyzed immediately or 24 h after incubation at 37°C. Thirty microliters were deposed on freshly cleaved mica (Veeco/Digital Instruments), left at room temperature for 5 min, washed with 5 ml of ddH_2O and dried under nitrogen flow. Measurements were carried out on a Multimode AFM with a Nanoscope V system operating in tapping mode, using standard antimony-doped silicon probes (T: 3.5–4.5 μm, L: 115–135 μm, W: 30–40 μm, K: 20–80 N/m, Bruker) with a scan rate in the 0.5–1.2 Hz range, proportional to the area scanned. For each sample five distinct regions were scanned and AFM images were analysed with the Scanning Probe Image Processor (SPIP, version 5.1.6, release 13 April 2011, www.imagemet.com) data analysis. To minimise possible artefacts, freshly cleaved mica and freshly cleaved mica soaked with 10 mM PB were used as controls.

Transmission electron microscopy (TEM). TEM was used to investigate the structure of p17, diluted at 4 μM in 10 mM PB, pH 7.4, before and 24 h after incubation at 37°C. Ten microlitres of p17 was dropped onto 300-mesh Formvar/carbon nickel grids (Electron Microscopy Science), and after 5 min the solution was removed. Samples were counterstained for 5 min with a saturated solution of uranyl acetate, washed with MilliQ water to eliminate excess uranyl acetate, and allowed to air dry. TEM analyses were performed with a Libra 120 apparatus operating at 120 kV equipped with a Proscan Slow Scan CCD camera (Carl Zeiss).

Binding to hydrophobic fluorescent probe. The hydrophobicity and conformational changes of p17 were evaluated using the hydrophobic fluorescent dye 8-anilinonaphthalene sulphonic acid

(ANS, Molecular Probes Invitrogen) and 4,4′-dianilino-1,1′-binaphthyl-5,5′-disulfonic acid (Bis-ANS, Sigma-Aldrich) [2]. In particular, fluorescence titrations were performed by adding small aliquots of ANS or Bis-ANS (from 0 up to 500 μM from 4 mM stock solution) to 4 μM freshly diluted p17 before (time 0) and after 24 h incubation at 37°C. Emission spectra were recorded at 25°C, between 400 and 600 nm and exciting samples at 350 nm using an Infinite M200 spectrofluorimeter (Tecan, Männedorf, Switzerland). Binding curves were obtained by plotting changes in fluorescence intensity, corrected for dilution effects and for the fluorescence of free ligand without the protein, as a function of ANS concentration. All binding data reported correspond to the average data obtained from two independent titrations.

Circular dichroism (CD) spectroscopy. Secondary structure of p17, myr-p17, $p17_{30-55}$ and $p17sc_{30-55}$ was determined by CD analysis. To this end p17 was analysed immediately after dilution to a final concentration 4 μM in 10 mM PB, pH 7.4, or different times (1–48 h) after incubation at 37°C. $p17_{30-55}$ and $p17sc_{30-55}$ were diluted to a final concentration of 50 μM in 10 mM PB (pH 7.4) and analyzed immediately before or 24 h after incubation at 37°C. The CD spectra were recorded on a Jasco J-815 spectropolarimeter (Jasco, Easton, MD) at 25°C from 200 nm to 260 nm (1.0 nm bandwidth and 0.1 nm resolution) using a 0.1 cm path length quartz cell. A sensitivity of 100 millidegrees, response of 4 s, scan speed of 50 nm/min and 5 accumulations were used. CD spectra are expressed as mean molar ellipticity (Φ, deg cm^2 $dmol^{-1}$) as a function of wavelength after subtracting those generated from PB alone. Each analysis was performed in triplicate, at least.

Nuclear magnetic resonance (NMR) spectroscopy studies. This technique has already been employed to investigate the ability of several natural and syntethic ligands, as well as tetracycline, to interact with oligomeric assemblies of different amyloidogenic proteins [3]. NMR spectra were

acquired on a Bruker AVANCE III 600 MHz NMR spectrometer equipped with a QCI (^{1}H, ^{13}C, ^{15}N/^{31}P and ^{2}H lock) cryogenic probe. The samples were prepared as follows: the stock solution of p17 (1.56 mg/ml, 91 µM) in PB containing 0.5 M NaCl, pH 6, was diluted to 25 µM in the NMR sample; the stock solution of 4 mM tetracycline in D_2O was diluted to 2 mM in the NMR sample; D_2O was added to reach the desired volume (560 µL). The pH of each sample was measured with a microelectrode (Mettler Toledo) for 5 mm NMR tubes and adjusted to pH 7.2 with NaOD and/or DCl. All pH values were corrected for isotope effect. The acquisition temperature was 25°C. 1D ^{1}H-NMR spectra were recorded (zgesgp pulse sequences in Bruker library) with 128 scans, spectral width of 12 ppm, relaxation delay of 2 s. 1D STD-NMR spectra were recorded (stddfiffesgp.3 pulse sequences in Bruker library) with 1024 scans, spectral width of 12 ppm, saturation times of 2 s, 1.5 s, 1 s, 0.65 s, 0.35 s. They were processed with a line broadening of 0.3 Hz and corrected for phase and baseline. The samples were co-incubated for different times ranging from t = 0 (experiment run just after sample preparation) to 144 hours.

Effect of tetracycline on the sensitivity of p17 to protease digestion. p17 at a final concentration of 4 µM in 1 M PB, pH 7.4, was digested with trypsin (Merck, Darmstadt, Germany, 1:0.03 w/w peptide to enzyme ratio) at 37°C for 30 min in the absence and presence of tetracycline (1:0.25 and 1:1 w/w peptide-to-drug ratio). The reaction was stopped by placing samples at 4°C for 10 min [4]. Each sample was then diluted in sample buffer (4x), electrophoresed on 16% Tris–Tricine SDS–PAGE gel under reducing conditions and stained with 0.05% Coomassie blue solution. The mean density of the positive Coomassie bands at 17 kDa were analysed using Quantity One software (Bio-Rad). The extent of proteolysis was calculated as the percentage of resistant peptide compared to the total amount.

Behavioral testing on rodents. *Sensorimotor responses:* Visual reflex and posterior legs extension reflex were measured by holding the animal by its tail and slowly lowering it towards a black surface. Motor coordination and equilibrium were assessed by the distance covered and the latency to fall off a horizontal wooden rod and a metal wire rod. Prehensility and motor coordination were measured as the distance covered on the wire hang test, which consisted of allowing the animal to cling from the middle of a horizontal wire (2 mm diameter x 40 cm length) with its forepaws for two trials of 5 s and a third 60 s trial.

Corner test: Neophobia to a new home-cage was assessed by introducing the animal into the centre of a standard square cage (Macrolon, 35 x 35 x 25 cm) with fresh bedding and counting the number of corners visited and rearings during a period of 30 s. The latency of the first rearing was also recorded.

Open field test: Mice were placed in the centre of the apparatus (home-made, wooden, white, 55 x 55 x 25 cm high) and observed for 5 min. Patterns of horizontal locomotor activity (distance covered and thigmotaxis) and vertical movement (rearings) were analyzed throughout the test. Initial freezing, self-grooming behavior and the number of urine spots and defecation boli were also recorded.

Dark and light box test: Anxiety-like behavior was measured in a dark-light box. The apparatus consisted of two compartments (black: 27 x 18 x 27 cm with a red light; white: 27 x 27 x 27 cm with white lighting intensity of 600 lux) connected by an opening (7 x 7 cm). The mice were introduced into the black compartment and observed for 5 min. The latency to enter the lit compartment, the time spent in the lit compartment and the number of rearings were recorded.

Boissier's four hole-board test: Exploratory behavior was measured as the number of head-dips and time spent head-dipping on each of the four holes (3 cm diameter) equally spaced in the floor of the

hole-board (woodwork white box of 32 x 32 x 32 cm). The latencies of movement, first dipping and four hole dipping were recorded.

Tail suspension test: Mice were suspended by the tail to assess depression-like behavior. The mouse was suspended 30 cm above the surface. The tail was fixed with adhesive tape at 1 cm from its tip. The duration of immobility (defined as the absence of all movement except for those required for respiration) was scored during 6 min.

Novel object recognition (NOR) test: Animals were placed in the middle of a black maze with two arms angled 90º, each measuring 25 cm x 5 cm. The 20 cm high walls could be lifted off for easy cleaning. The lighting intensity was 30 lux. The objects to be discriminated were made of wood (5-6 cm high, brightly colored). After two previous days of habituation, the animals were submitted to a 10 min acquisition trial (first trial) during which the mouse was placed in the maze in the presence of two identical novel objects (A + A) placed at the end of each arm. A 10 min retention trial (second trial) occurred 2 h later, replacing object A’ in the maze by object B (A + B). Another 10 min retention trial (third trial) occurred 24 h later, replacing object A in the maze by object C (B + C). The time that the animal explored the new object and the old object were recorded. In order to avoid object preference biases, the sequence of presentation of the different objects was counterbalanced in each experimental group. The maze and the objects were cleaned with 96° ethanol between different animals, so as to eliminate olfactory cues.

Morris water maze test: Animals were tested for spatial learning and memory in the Morris water maze (MWM), consisting of 1 day of cue learning and 6 days of place task learning for spatial reference memory, followed by one probe trial. To test the spatial learning acquisition, mice were trained to locate a hidden platform (10 cm diameter, located 20 cm from the wall and 0.5 cm below the water surface) in a circular pool (100 cm diameter, 40 cm height, 25ºC opaque water, surrounded by black curtains) by relying on distinctive landmarks as visual cues (four trial sessions

of 60 s per day) [5]. On day 7, after one trial of place learning, the platform was removed and the mice performed a probe trial of 60 s to test the retention of learning. A computerized tracking system (SMART, Panlab S.A., Barcelona, Spain) allowed to measure the distance covered during the learning tasks, along with the time spent in each quadrant of the pool after the removal of the platform in the probe test.

Histological analysis. Immunohistological analysis were carried out on human and murine brains to determine the localization of p17. Human samples, fixed in 4% paraformaldehyde in 100 mM PB and paraffin, were processed according to standard histological protocols.

After completion of the behavioral tests, at 1 month after injection, mice were anesthetized and transcardially perfused with 100 mM PB, pH 7.4, containing 0.1 mg/mL heparin (Mayne Pharma, Spain) followed by 4% paraformaldehyde in 100 mM PB. The brains of six animals per group were subjected to sectioning throughout the whole bregma and examined every 10th section. Murine brains were then post-fixed overnight in cold 4% paraformaldehyde in 100 mM PB, rinsed with cold 100 mM PB, then dehydrated in a graded ethanol series, cleared in xylene and embedded in paraffin.

Sections were incubated at room temperature over night with the monoclonal anti-p17 MBS-3 antibody (1:100 vol/vol), the mouse monoclonal anti-CD105 antibody (Abcam, 1:100 dilution vol/vol) recognizing activated microvessels or the rabbit polyclonal anti- CD105/endoglin antibody (Abcam, 1:100 dilution vol/vol), anti-CD68 antibody (Abcam125212; 1:100 dilution vol/vol), rabbit polyclonal anti-CD31 antibody (Abcam, 1:50 dilution vol/vol), the rabbit polyclonal anti-β-amyloid antibody (Abcam 2539, 1:100 dilution vol/vol), or the rabbit polyclonal anti p-tau antibody (Abcam151559, 1:100 dilution vol/vol). Sections were washed three times with 10 mM PBS, pH 7.4 and then incubated for 1h with the appropriate secondary antibodies: rabbit anti-mouse

biotinylated (Vector Labs; 1:500 dilution vol/vol), biotinylated-ABC Alexa Fluor (Invitrogen, dilution 1:50 vol/vol) , the goat anti-rabbit IgG (H&L Alexa Fluor® 647, 1:300 dilution vol/vol), the goat anti-mouse IgG (H&L Alexa Fluor® 488, 1:300 dilution vol/vol), 3,3' diaminobenzidine (DAB, Vector Labs, UK) or Nickel DAB (N-DAB, Vector Labs, UK) for immunohistochemistry. Nuclei were visualized by 4',6-diamidino-2-phenylindole (DAPI). Images were captured with Nikon 80i Digital Microscope using Nis Elements 3.21 software with multichannel capture option. Negative control slides were included where the primary antibody was replaced with 5 mM PBS, pH 7.4.

SUPPLEMENTARY FIGURES

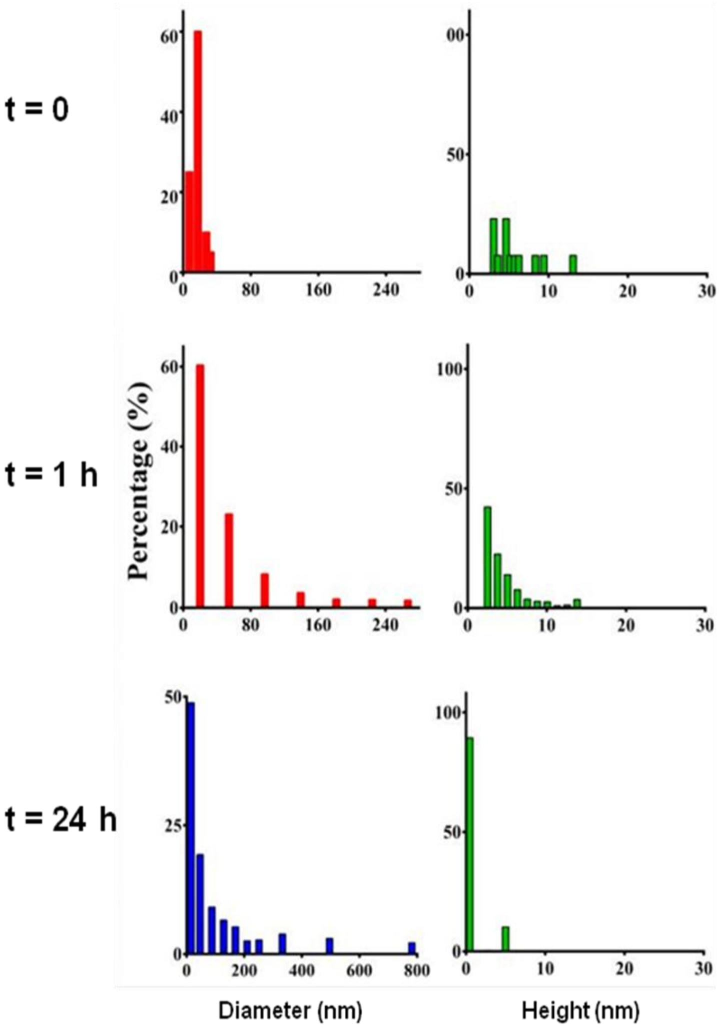

	t = 0	t = 1 h	t = 24 h
Diameter (nm)	7–35	20–96	–
Height (nm)	3–11	4–12	1–5
Length (nm)	–	–	49–250

Supplementary Figure S1. SPIP analysis of p17. Diameter and height distribution obtained from the SPIP analysis performed on the AFM images recorded on freshly diluted p17 (t = 0) at 4 μM in 10 mM PB (pH 7.4), or 1 h (t = 1 h) and 24 h (t = 24 h) after incubation at 37°C. Cumulative frequency graphs are reported. Diameter, heights and lengths obtained from the SPIP analysis performed on a minimum of 5 different areas are reported in the table.

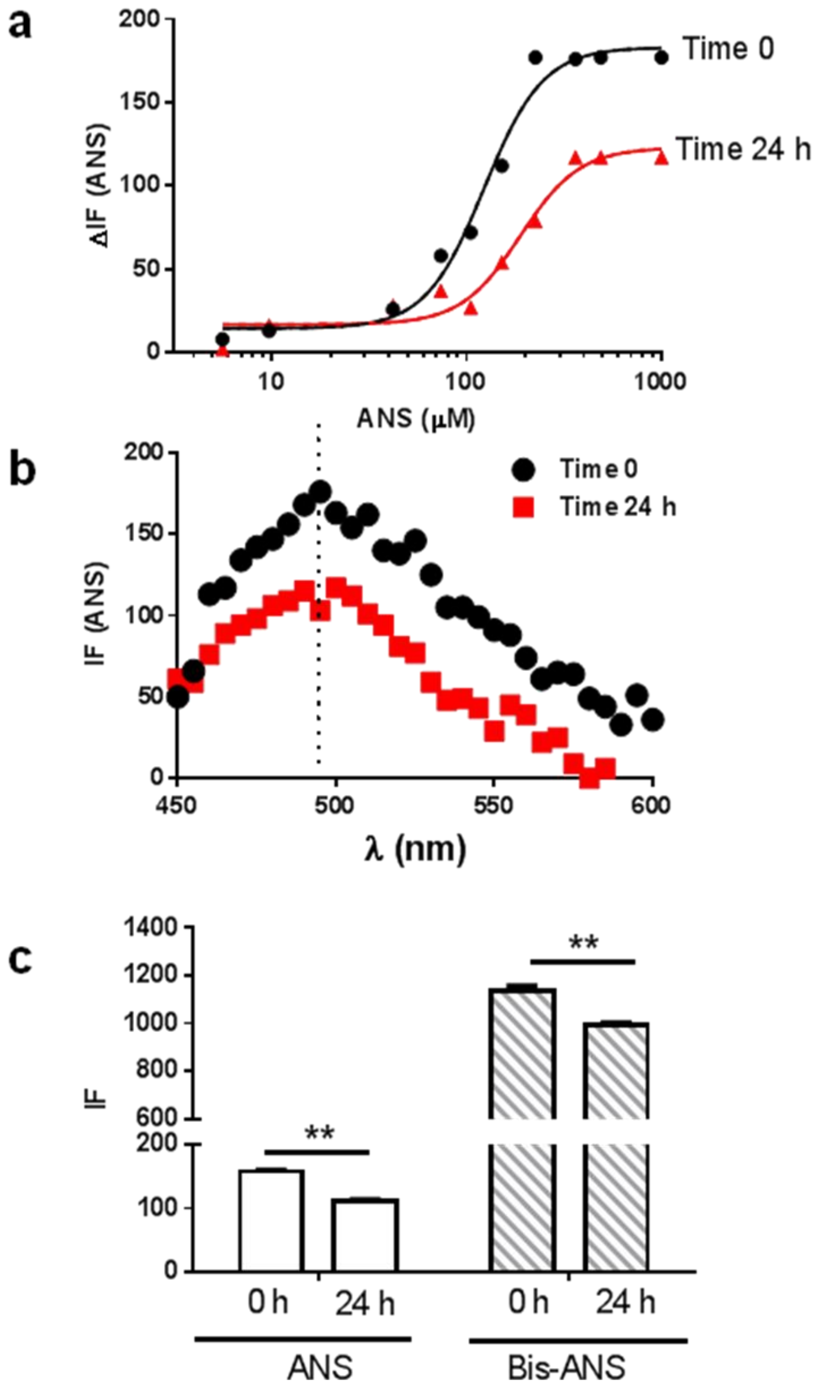

Supplementary Figure S2. Binding of fluorescent hydrophobic probes. (**a)** Concentration-dependence of ANS binding to 4 µM p17 before (time 0) and 24 h after incubation at 37°C (time 24 h). The lines represent the best-fitting curve of data. (**b)** ANS fluorescence emission spectra of 4 µM p17 before and 24 h after incubation at 37°C. (**c)** Binding of ANS or Bis-ANS to the p17 assemblies before (time 0) and 24 h after incubation at 37°C. Mean ± SD, n = 4. ***P* < 0.01, one-way ANOVA and Bonferroni *post hoc* test. FI, arbitrary unit of fluorescence intensity.

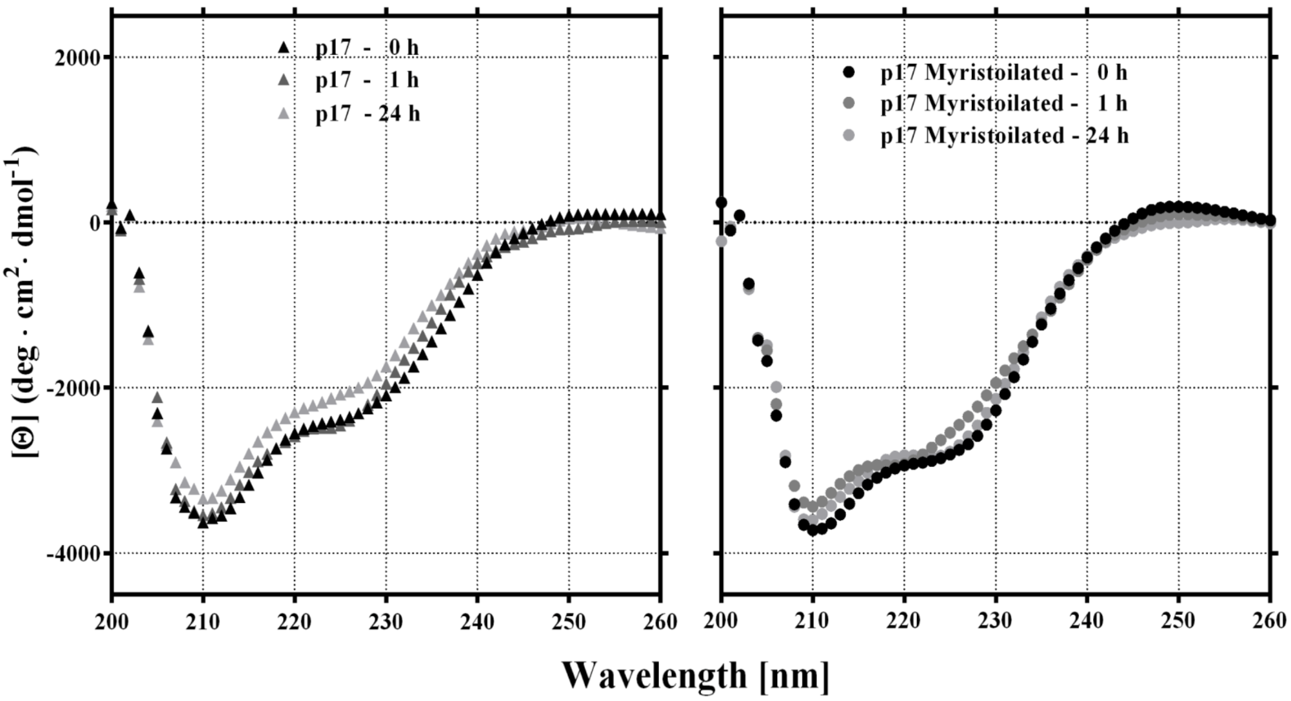

Supplementary Figure S3. Circular dichroism (CD) analysis of p17 and myr-p17. Freshly diluted 4 µM solutions of p17 and myr-p17 were prepared in 10 mM PB, pH 7.4. CD spectra were recorded immediately after diluting the sample (t = 0), 1 h (t = 1 h) and 24 h (t = 24 h) after incubations at 37°C. All measurements were performed at 37°C and 5 accumulations were used. CD spectra are expressed as mean molar ellipticity (Φ). At all of the time points considered p17 and myr-p17 had a similar conformation.

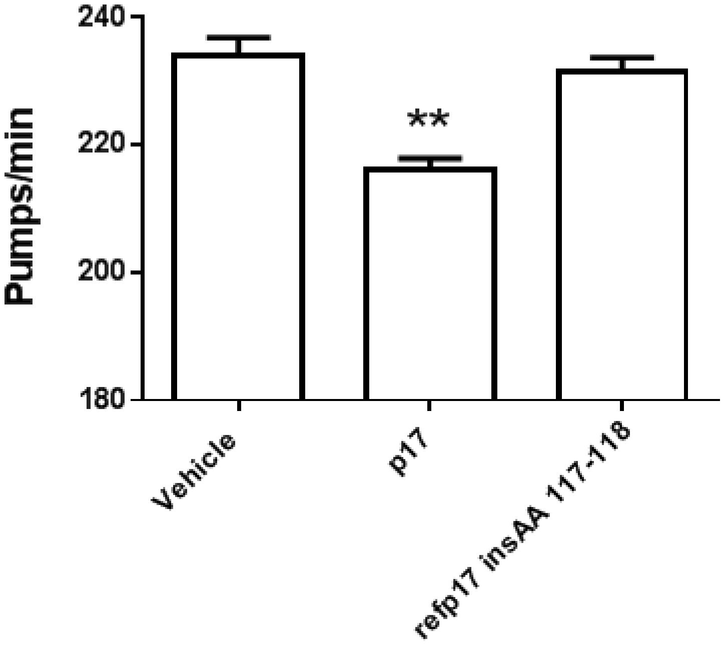

Supplementary Figure S4. Effect of unfolded p17 on pharyngeal pumping. N2 nematodes (100 worms/100 µL) were incubated for 2 h with 4 nM p17 or unfolded refp17 insAA 117–118 in the absence of OP50 *E. coli*. Nematodes were then plated on NGM plates seeded with bacteria and the pharyngeal pumping was scored 2 hours after plating. Control worms were fed 10 mM PB (pH 7.4) (vehicle). Data are expressed as the mean ± SE (n = 20 worms/group). ***P*< 0.01 *vs.* vehicle according to one-way ANOVA and Bonferroni *post hoc* test.

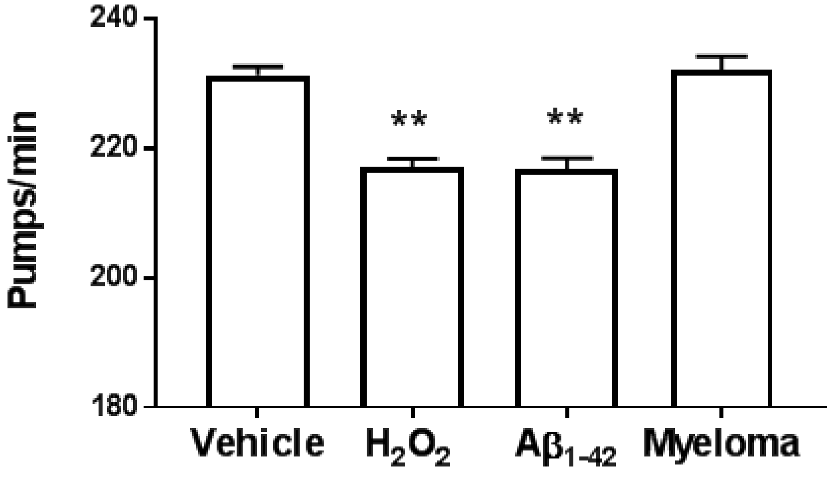

Supplementary Figure S5. Positive and negative controls on pharyngeal pumping. N2 nematodes (100 worms/100 µL) were incubated for 2 h with 10 mM H_2O_2, 3 µM oligomeric synthetic A$\beta_{1\text{-}42}$ or 100 µg/mL non-amyloidogenic immunoglobulin light chain purified from a patients suffering from myeloma (Myeloma) in the absence of OP50 *E. coli*. Nematodes were then plated on NGM plates seeded with bacteria and the pharyngeal pumping was scored 2 hours after plating. Control worms were fed 10 mM PB, pH 7.4 (Vehicle). Data are expressed as the mean ± SE (n = 30 worms/group). ***P*< 0.01 *vs.* vehicle according to one-way ANOVA and Bonferroni *post hoc* test.

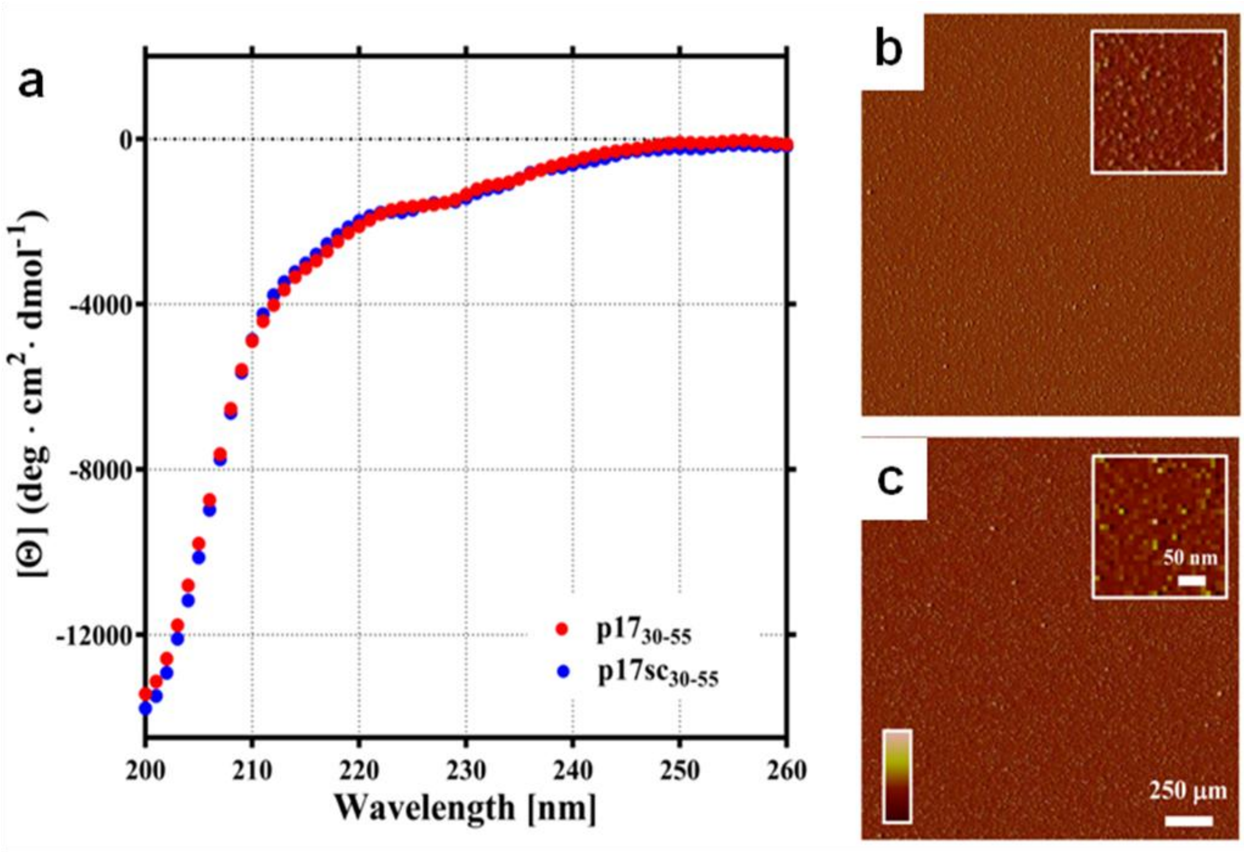

Supplementary Figure S6. Circular dichroism (CD) and Atomic force microscopy (AFM) analysis of p17$_{30\text{-}55}$ and p17$_{30\text{-}55}$ scrambled (p17sc$_{30\text{-}55}$) peptides. (a) Solutions of 50 µM p17$_{30\text{-}55}$ and p17sc$_{30\text{-}55}$ peptides were prepared in 10 mM PB, pH 7.4. CD spectra were recorded at 37°C 24 h after incubation and five accumulations were used. CD spectra are expressed as mean molar ellipticity (Φ). At all time points p17$_{30\text{-}55}$ and p17sc$_{30\text{-}55}$ showed a similar conformation. (**b**) p17$_{30\text{-}55}$ and (**c**) p17sc$_{30\text{-}55}$, at 50 µM in 10 mM PB, pH 7.4, were adsorb on freshly cleaved mica. AFM images were obtained in taping mode 24 h after incubation at 37°C and reported as amplitude data (Z range: -10/ +50 mV). Similar images were obtained at the other times points considered. Scale bar = 250 µm, inset = 50 nm.

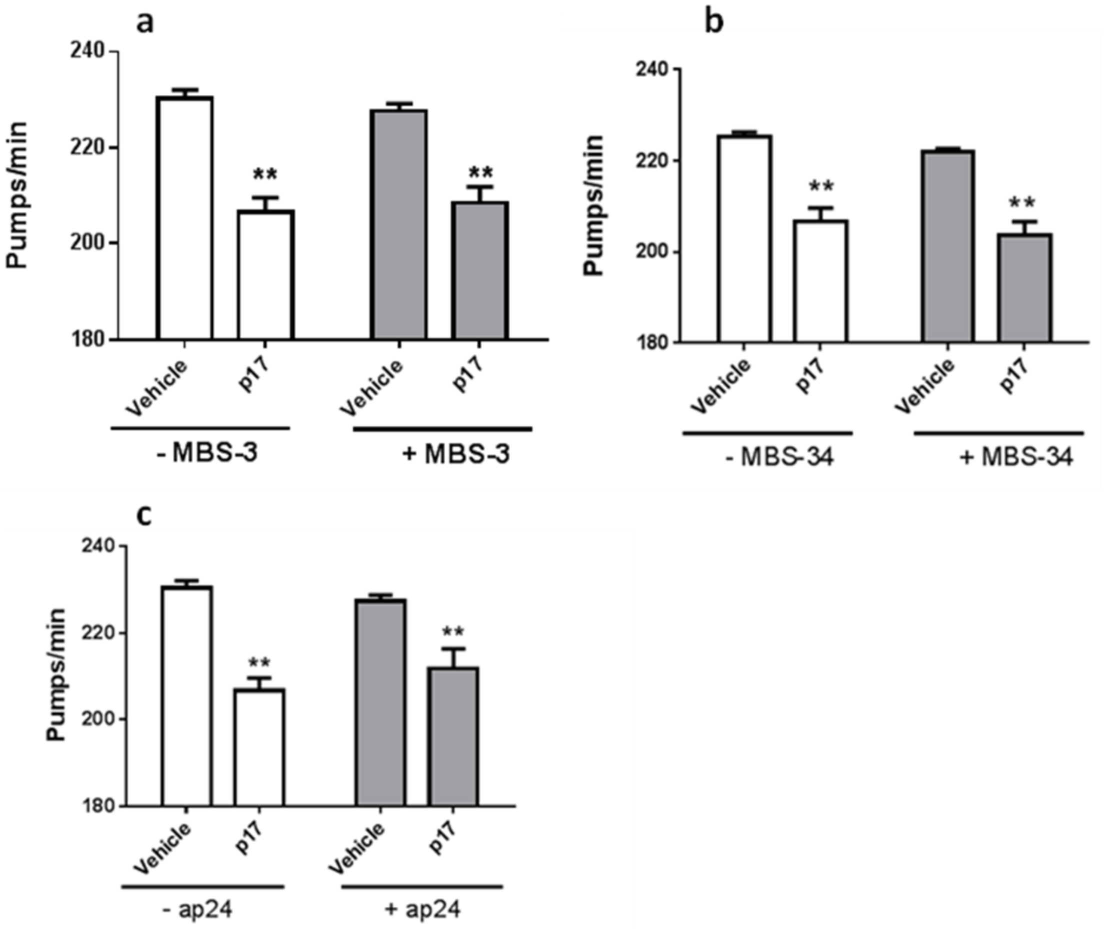

Supplementary Figure S7. Effect of MBS-3, MBS-34 and ap24 antibodies on the p17-induced pharyngeal impairment. Antibodies at 6 ng/µL in 10 mM PB (pH 7.4) were co-incubated with 4 nM p17 for 30 min at room temperature. Nematodes (100 worms/100 µL) were then fed with these solutions for 2 h in the absence of OP50 *E. coli* before plating on NGM plates seeded with bacteria. The pumping rate was scored 2 h after plating. Antibody alone (6 ng/µL) and 10 mM PB (pH 7.4) were used as positive and negative controls, respectively (vehicle). Data are expressed as the mean ± SE (n = 20 worms/group). $^{**}P < 0.01$ *vs.* vehicle according to two-way ANOVA and Bonferroni *post hoc* test.

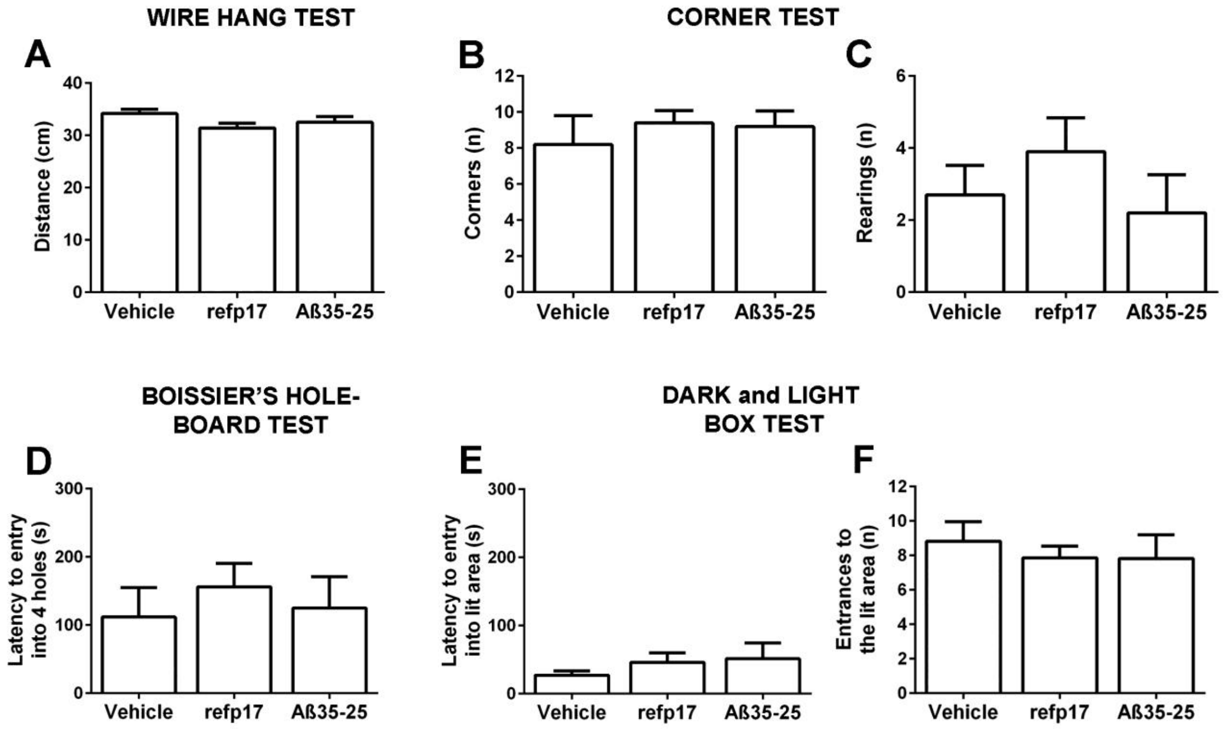

Supplementary Figure S8. The intrahippocampal injection of refp17 insAA 117–118 (refp17) and amyloid β 35-25 peptide (Aβ35-25) did not affect mice non-cognitive behavior. Bilateral deliveries of 1 µL of protein solution (0.5 µg/µL in artificial Cerebrospinal fluid (aCSF)) or aCSF alone (Vehicle). (**a**) refp17 and Aβ35-25 injected mice showed similar motor coordination in the wire hang test in respect to Vehicle animals. **(b-f)** refp17 and Aβ35-25 injected mice did not show behavioral and psychological symptoms of dementia–like as indicated by their similar responses than Vehicle animals. Namely, they showed absence of neophobia in **(b)** number of corner explorations and (**c**) number of rearings in the corner test, exploratory curiosity indicated by (**d**) the latency to explore the whole 4 holes in the Boissier's hole-board test, and absence of anxiety indicated by (**e**) latency to enter to the lit compartment and (**f**) number of explorations in the dark and light box test. Mean ± SE (n = 6-7 mice/group). No statistical differences (one-way ANOVA).

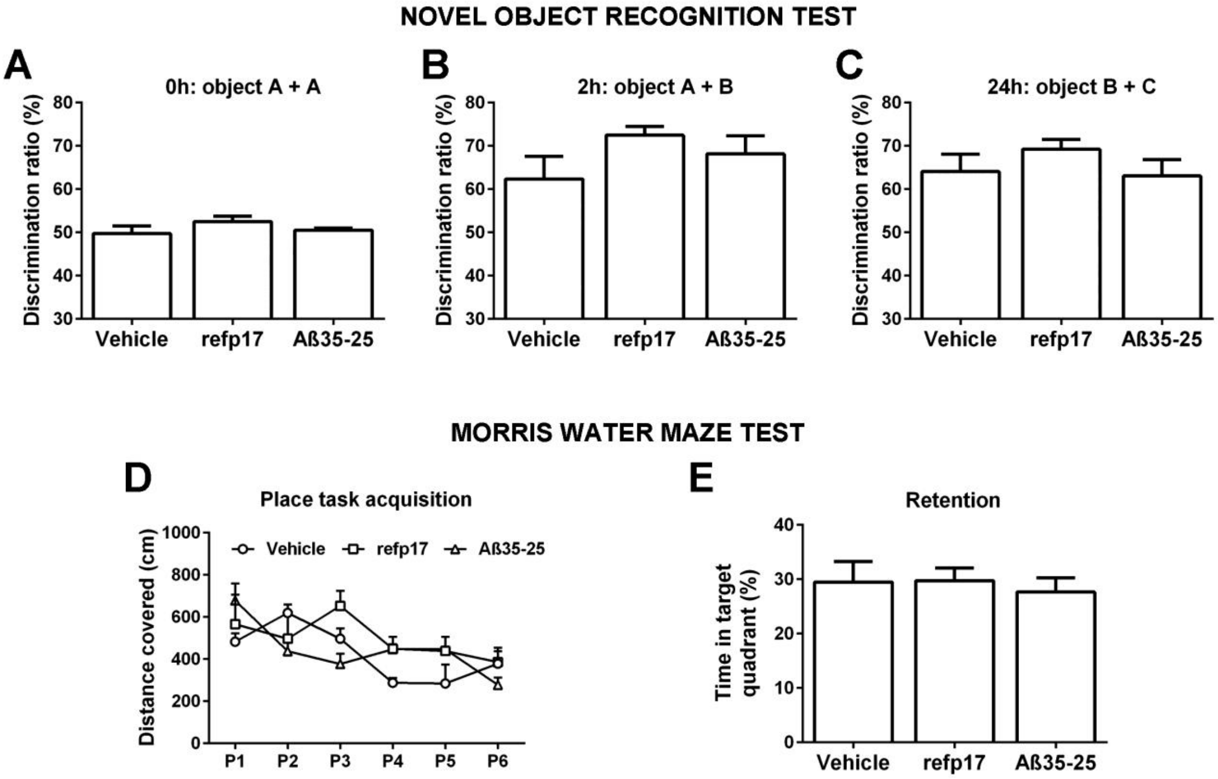

Supplementary Figure S9. The intrahippocampal injection of refp17 insAA 117–118 (refp17) and amyloid β 35-25 peptide (Aβ35-25) did not affect mice learning and memory. Bilateral deliveries of 1 µL of protein solution (0.5 µg/µL in artificial Cerebrospinal fluid (aCSF)) or aCSF alone (Vehicle). Cognition was tested with the novel object recognition test (NOR) and Morris water maze test (MWM). (**a-c**) In the NOR, refp17 and Aβ35-25 injected mice discriminated the novel object from the familial one, in the same way as Vehicle animals, at time 0 h **(a)**, 2h **(b)** and 24 h **(c)**. (**d-e**) In the MWM, refp17 and Aβ35-25 mice did not show deficiencies in spatial learning and memory. **(d)** refp17 and Aβ35-25 mice reduced the distances throughout six days of training to find the position of a hidden scape platform, similarly to Vehicle mice. (**e**) refp17 and Aβ35-25 mice spent more time swimming in the pool quadrant where the platform had been located when the platform was removed for the probe trial of retention, similarly to Vehicle mice. Mean ± SE (n = 6-7 mice/group). Statistical analysis showed no differences between the groups of mice in (**a-c**) and (**e**) (one-way ANOVA) and a similar acquisition of learning for the three groups in (**d**) (two-way repeated ANOVA measures).

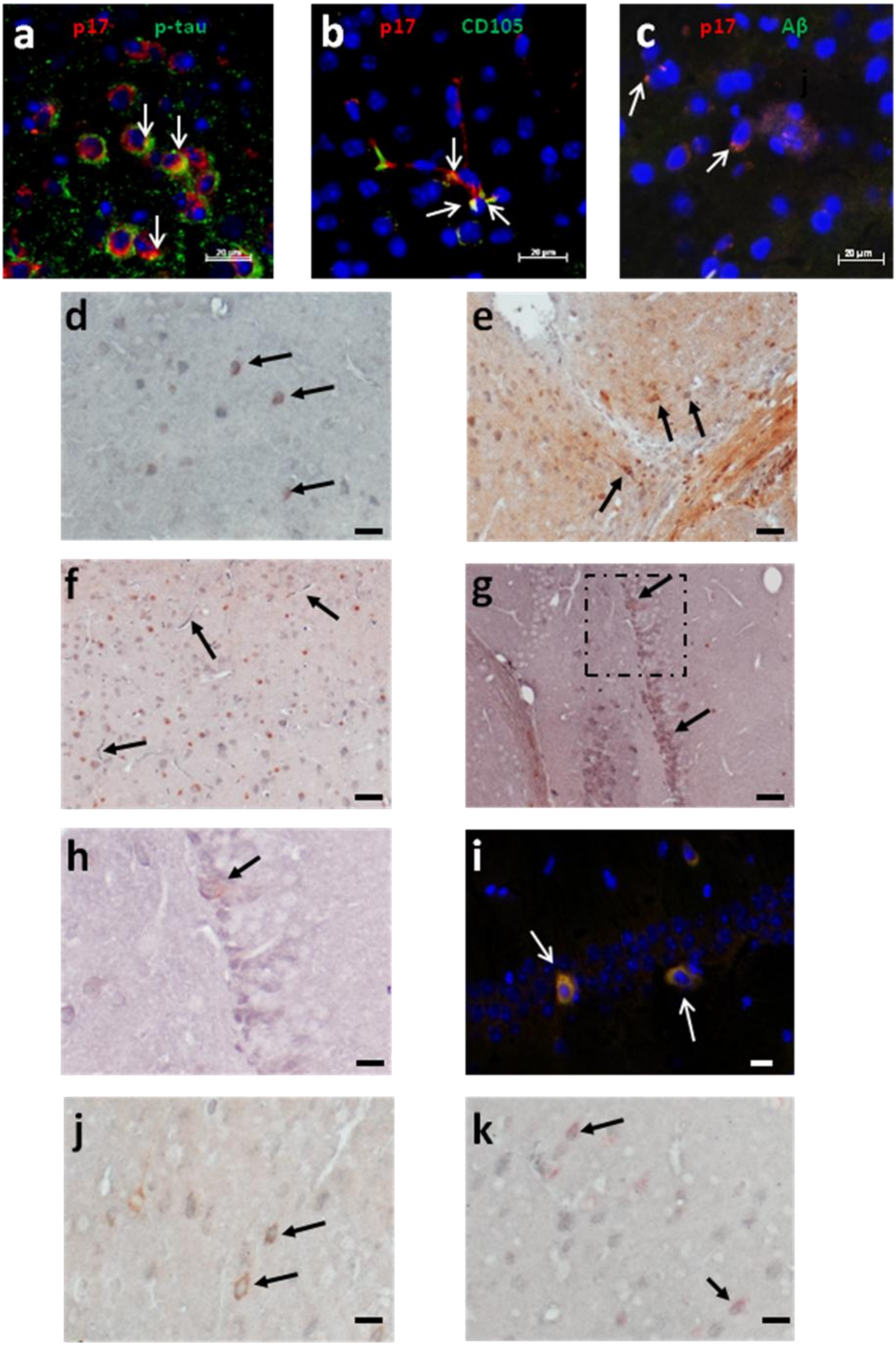
a
p17
p-tau
20 μm
b
p17
CD105
20 μm
c
p17
Aβ
20 μm
d
e
f
g
h
i
j
k

Supplementary Figure S10. Regional and direct co-localisation of p17 with tau/Aβ and CD105 in the brain of mice 1 month after p17 injection. (**a-c**) Representative immunofluorescent images of brain sections fixed and stained for p17 (red), p-tau (green), CD105 (green) or Aβ (green). Nuclei were counter-stained with DAPI (blue). Arrows indicate the yellow/orange signal showing the direct co-localization of p17 with (**a**) p-tau in cortical neurones and (**b**) CD105 staining in cortical microvessels. (**c**) Arrows indicate the cortical plaque-like Aβ-positive staining which, although weak, co-localised with p17. Scale bars= 20 μm. (**d-h**) Representative immunohistochemistry of brain sections showing the staining for p17 (N-DAB stain, gray-black) and the peri-nuclear staining for p-tau (DAB stain, brown) in cortical neurones (**d-e**), (**f**) their regional localization in cortical neurones and microvessels and (**g**) in hippocampal neurones, which can be better appreciated in the enlarged area border by square dot line shown in panel **h**. Arrows show their co-localization. (**i**) Immunofluorescent staining showing yellow co-localisation of p17 (red) and p-tau (green) in hippocampal neurones. Nuclei were counter-stained with DAPI (blue). Scale bars= 50 μm in (**d-g**) and 10 μm in (**h-i**). (**j-k**) Representative immunohistochemistry showing co-localization between p17 (N-DAB stain) and β-amyloid (DAB stain) within cortical neurones and possibly developing plaques.

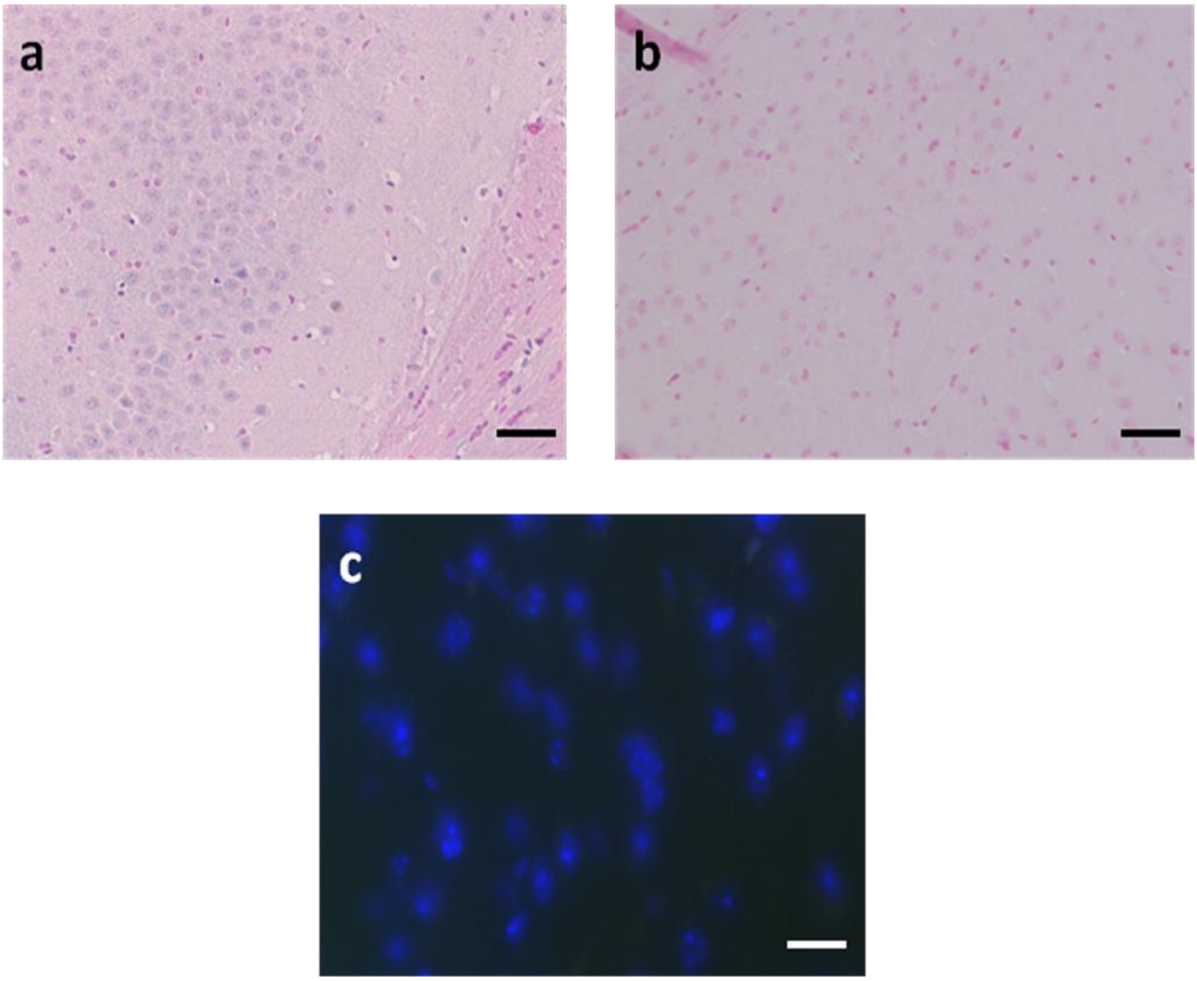

Supplementary Figure S11. Negative control staining of p17. (a-b) Representative immunohistochemical images of brain sections showing the negative control staining for p17 (N-DAB) in rat cortical brain tissue. (**a**) The p17 primary antibody and (**b**) the secondary antibody were replaced with 5 mM PBS, pH 7.4. (**c**) Negative control immunofluorescent image obtained by incubation of a brain section without the p17 primary antibody. Nuclei were counterstained with DAPI (blue). Scale bars= 50 µm.

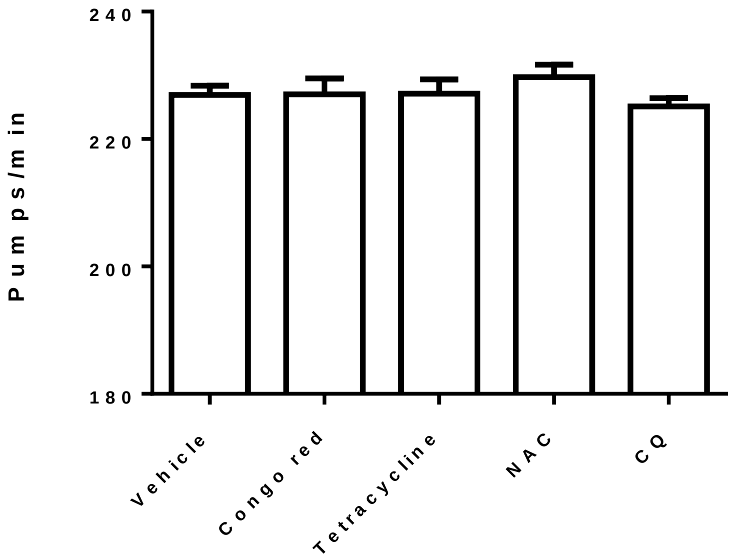

Supplementary Figure S12. Effect of drugs alone on pharyngeal pumping. N2 nematodes (100 worms/100 µL) were incubated for 2 h with 200 µM Congo red, 50 µM tetracycline, 5 mM *N*-acetylcysteine (NAC) or 25 µM clioquinol (CQ) in the absence of OP50 *E. coli*. Nematodes were then plated on NGM plates seeded with bacteria and the pharyngeal pumping was scored 2 h after plating. Control worms were fed only 10 mM PB, pH 7.4, (Vehicle). Data are expressed as the mean ± SE (n = 20 worms/group).

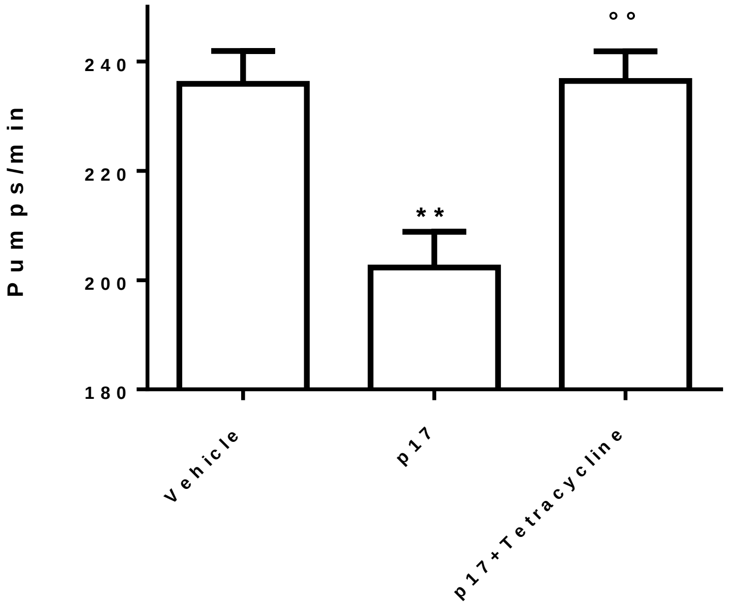

Supplementary Figure S13. Effect of tetracycline administered after nematode exposure to p17. N2 nematodes (100 worms/100 µL) were incubated for 2 h with 4 nM p17 (p17) and then treated with 50 µM tetracycline (p17+tetracycline) for an additional 30 min. Nematodes were then plated on NGM plates seeded with bacteria and the pharyngeal pumping was scored 2 h after plating. Control worms were fed 10 mM PB, pH 7.4 alone (Vehicle). Data are expressed as the mean ± SE (n = 30 worms/group). ***P* < 0.01 *vs.* vehicle and °°*P*< 0.01 *vs.* p17 according to one-way ANOVA and Bonferroni *post hoc* test.

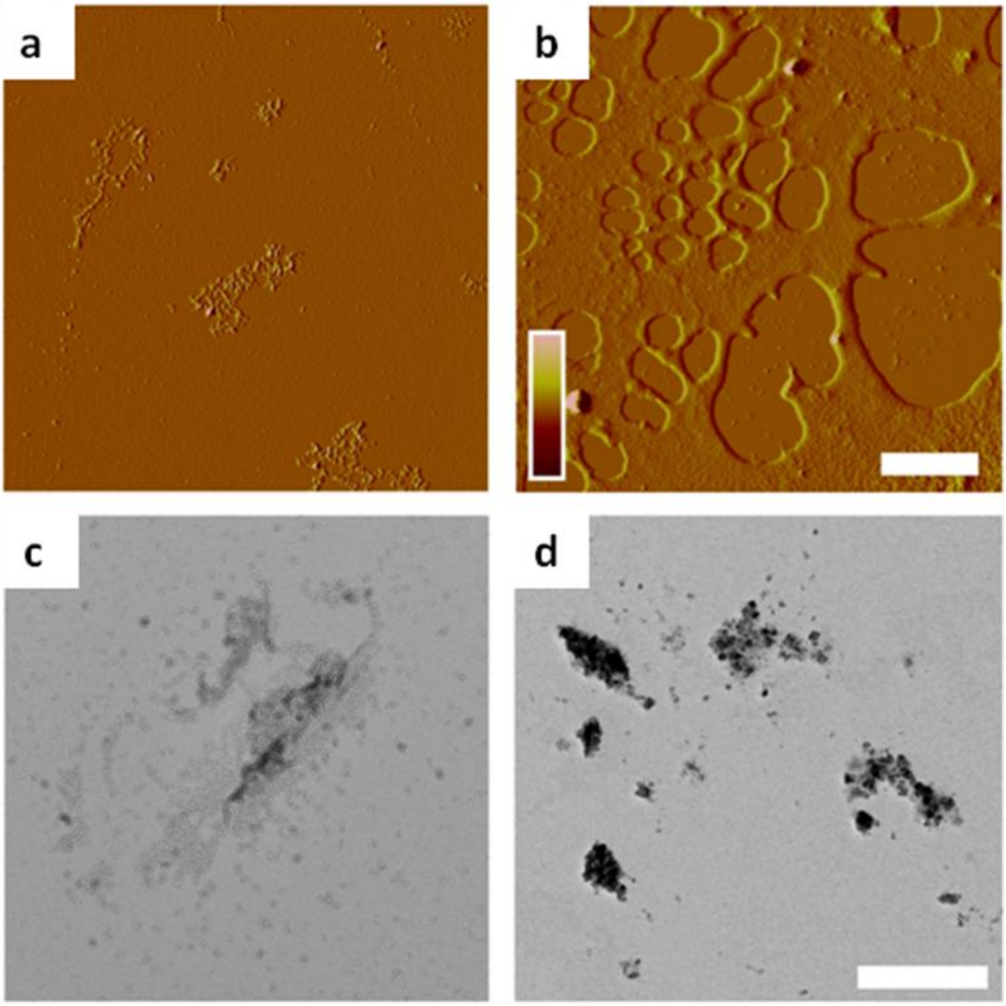

Supplementary Figure S14. Effect of tetracycline on p17 aggregation. Taping mode (**a-b)** AFM images (amplitude data) and (**c-d)** TEM micrographs. p17 at 4 μM in 10 mM PB, pH 7.4, was incubated at 37°C for 48 h (**a, c)** alone or (**b, d)** with tetracycline (1:2 molar ratio). The colored bar in AFM images corresponds to an amplitude range of − 50/ + 50 mV for panel (**a)** and − 180/ + 180 mV for panel (**b**). Scale bar: AFM = 2 μm, TEM = 1 μm. The TEM micrographs are representative of a minimum of 10 different areas.

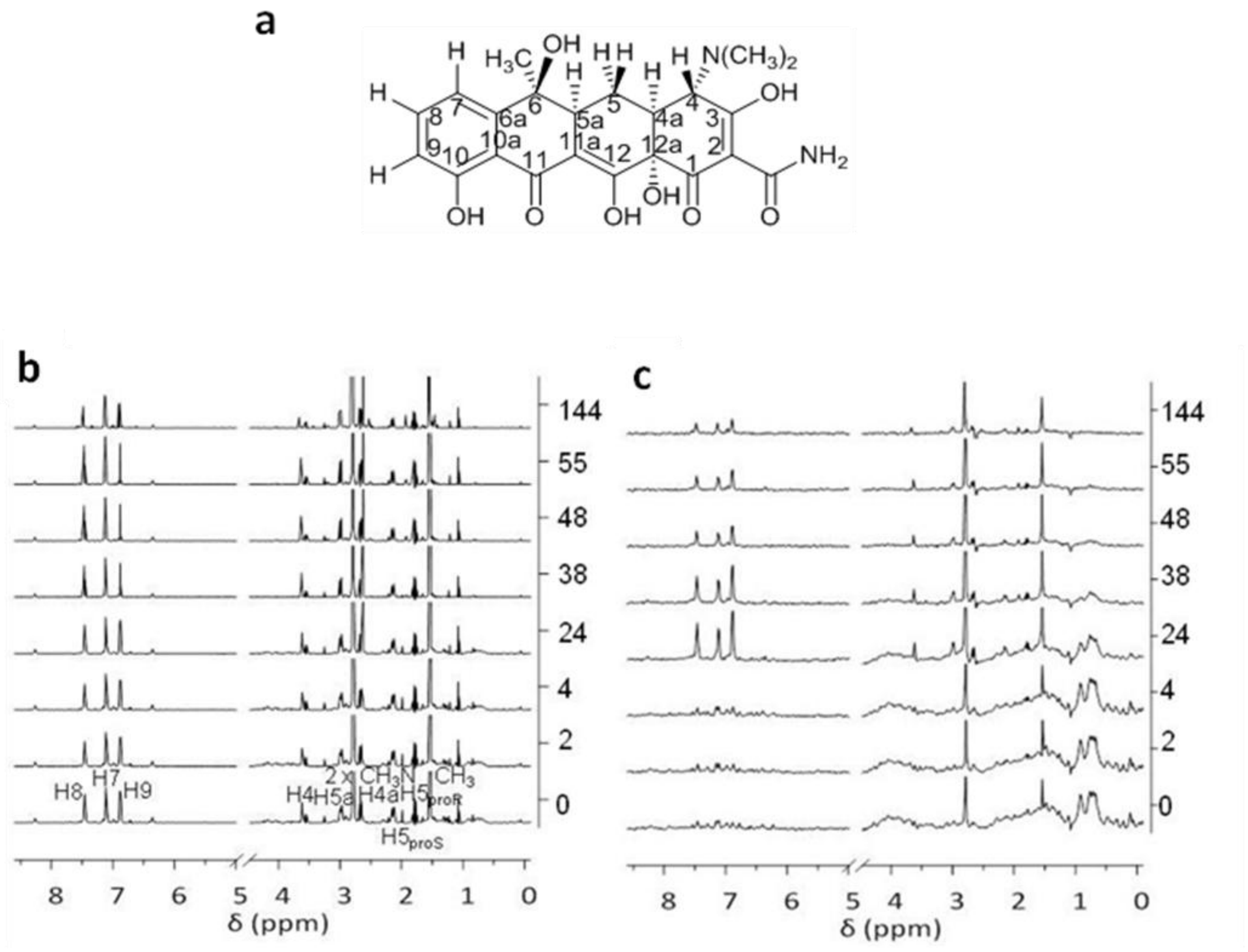

Supplementary Figure S15. NMR spectra. (**a**) Tetracycline structure and numbering. (**b)** 1H-NMR spectra of p17-tetracycline mixture at a 1:80 molar ratio were recorded after different incubations (from 0 up to 144 h, as indicated on the ordinate axis). NS = 128. (**c**) STD-NMR spectra of the mixture recorded at different protein-ligand co-incubation times (from 0 up to 144 h, as indicated on the ordinate axis). NS = 1024, saturation time = 2 s, on-resonance frequency = -1.0 ppm, off-resonance frequency = 40 ppm. All spectra were recorded at 25°C on the same sample. Tetracycline interacted with p17 in a time-dependent manner, as indicated by the strong increase of the tetracycline STD signal from 4 h up to 24 h after incubation with the protein.

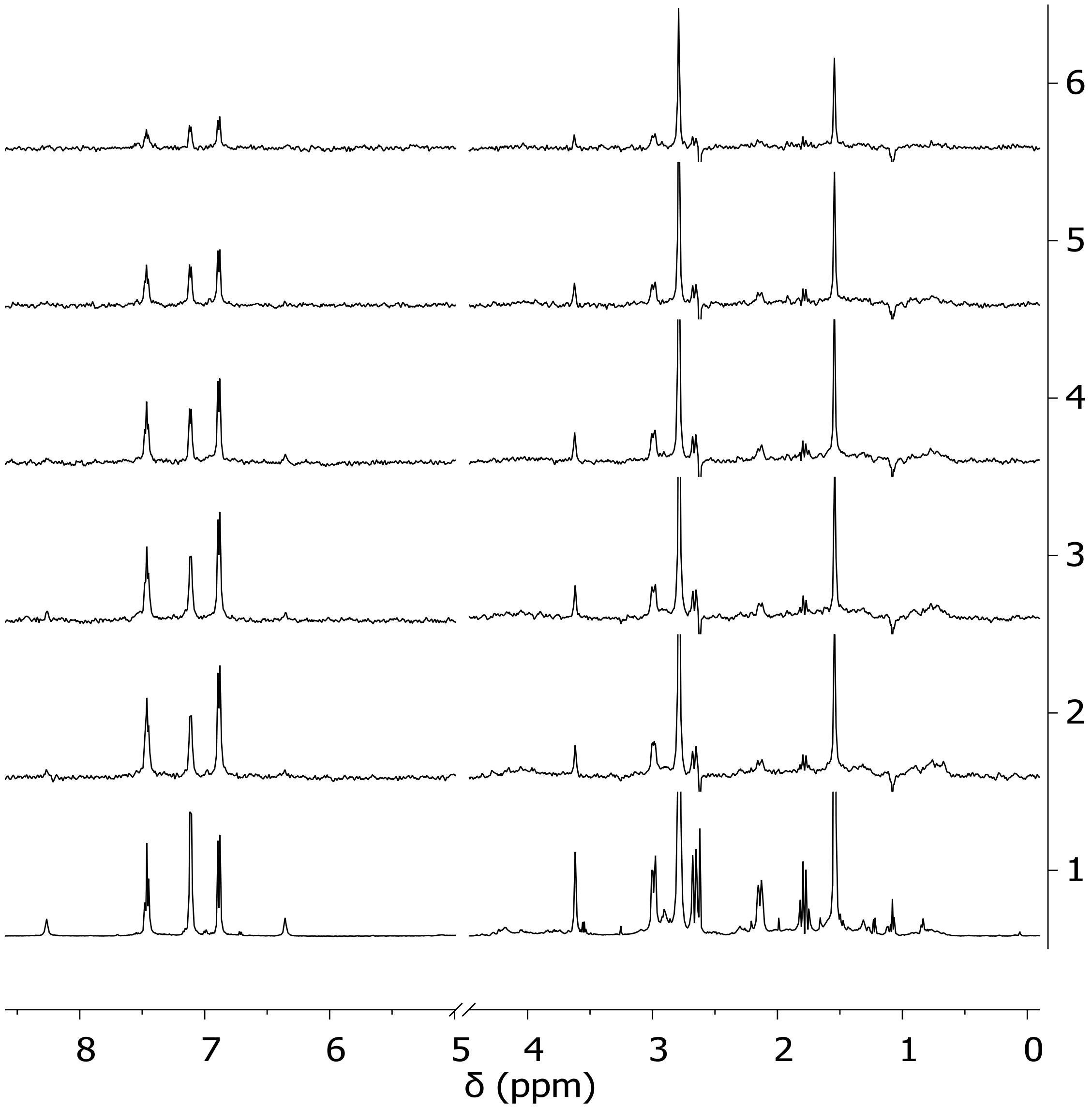

Supplementary Figure S16. NMR spectra. 1H-NMR spectrum of p17-tetracycline mixture at a 1:80 molar ratio (NS = 512; 2–6). STD spectra of the mixture recorded at different protein saturation times (1, 0 s; 2, 2 s; 3, 1.5 s; 4, 1 s; 5, 0.65 s; 6, 0.35 s). NS = 1024, on-resonance frequency = -1.0 ppm, off-resonance frequency = 40 ppm. Spectra 1–6 were recorded on the same sample at 25°C. A lowering of STD signals passing from spectra with 2 s saturation time to spectra with 0.35 s saturation time was observed.

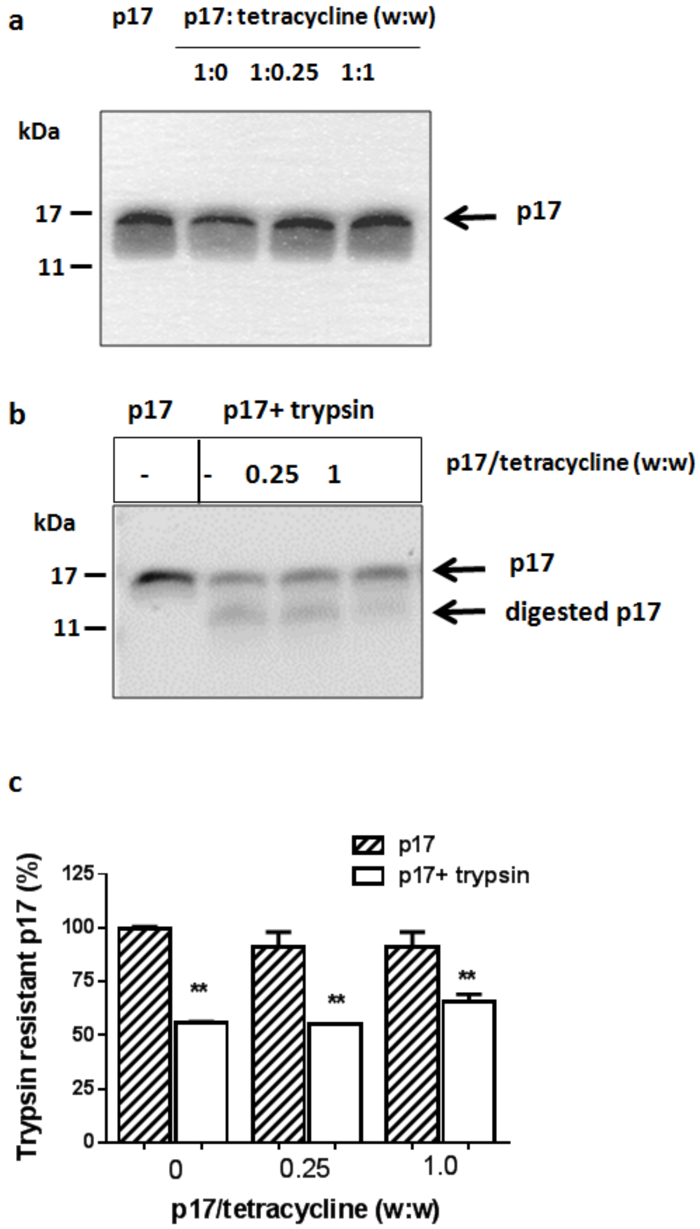

Supplementary Figure S17. Tetracycline did not affect p17 sensitivity to protease degradation. p17 at 4 μM, in 1 M PB, pH 7.4, was incubated at 37°C for 30 min: (**a)** alone or with different amounts of tetracycline; (**b)** alone, with trypsin (p17+trypsin, 1:0.03 w/w peptide-to-enzyme ratio) or with trypsin and tetracycline. Samples were then diluted in sample buffer, electrophoresed on 16% Tris–Tricine SDS-PAGE gels under reducing conditions and Coomassie stained. Arrows indicate the bands corresponding to p17 and the digested p17 formed after trypsin digestion (digested p17). (**c)** The mean density of the positive Coomassie bands at 17 kDa were analysed using Quantity One software (Bio-Rad) and the percentage of trypsin-resistant p17 was calculated. **$P \leq 0.01$ *vs*. p17, one-way ANOVA.

References

1 Diomede, L. *et al.* The new beta amyloid-derived peptide Abeta1-6A2V-TAT(D) prevents Abeta oligomer formation and protects transgenic C. elegans from Abeta toxicity. *Neurobiol Dis* **88**, 75-84, doi:10.1016/j.nbd.2016.01.006 (2016).

2 Messa, M. *et al.* The peculiar role of the A2V mutation in amyloid-beta (Abeta) 1-42 molecular assembly. *J Biol Chem* **289**, 24143-24152, doi:10.1074/jbc.M114.576256 (2014).

3 Airoldi, C. *et al.* Tetracycline prevents Abeta oligomer toxicity through an atypical supramolecular interaction. *Org Biomol Chem* **9**, 463-472, doi:10.1039/c0ob00303d (2011).

4 Forloni, G., Colombo, L., Girola, L., Tagliavini, F. & Salmona, M. Anti-amyloidogenic activity of tetracyclines: studies in vitro. *FEBS Lett* **487**, 404-407 (2001).

5 Garcia-Mesa, Y. *et al.* Oxidative Stress Is a Central Target for Physical Exercise Neuroprotection Against Pathological Brain Aging. *The journals of gerontology. Series A, Biological sciences and medical sciences* **71**, 40-49, doi:10.1093/gerona/glv005 (2016).
